# Interaction of hemorphins with ACE homologs

**DOI:** 10.1038/s41598-023-30771-0

**Published:** 2023-03-06

**Authors:** Amie Jobe, Priya Antony, Suhib Altabbal, Yusra Al Dhaheri, Ranjit Vijayan

**Affiliations:** 1 Department of Biology, College of Science, United Arab Emirates University, PO Box 15551, Al Ain, United Arab Emirates; 2 The Big Data Analytics Center, United Arab Emirates University, PO Box 15551, Al Ain, United Arab Emirates; 3 Zayed Center for Health Sciences, United Arab Emirates University, PO Box 17666, Al Ain, United Arab Emirates

**Keywords:** Virtual drug screening, Pharmacodynamics, Structure-based drug design

## Abstract

Hemorphins, short bioactive peptides produced by enzymatic cleavage of β-hemoglobin, exhibit antihypertensive properties by inhibiting angiotensin-1 converting enzyme (ACE1). ACE1 is a key player in the renin–angiotensin system (RAS) and regulates blood pressure. ACE1 and its homolog, ACE2, which exhibit opposing activities in the RAS, share considerable similarity in their catalytic domains. The primary objective of this study was to identify and contrast the molecular mechanisms underlying the interaction of hemorphins of camels and that of other mammals with the two ACE homologs. In silico docking and molecular dynamics simulations were performed for ACE1 and ACE2, along with in vitro confirmatory assays for ACE1. The C-domain of ACE1, primarily involved in regulating blood pressure, was used along with the N-terminal peptidase domain of ACE2. The findings revealed conserved hemorphin interactions with equivalent regions of the two ACE homologs and differential residue-level interactions reflecting the substrate preferences of ACE1 and ACE2 considering their opposing functions. Therefore, conserved residue-level associations and implications of poorly conserved regions between the two ACE receptors may potentially guide the discovery of selective domain-specific inhibitors. The findings of this study can provide a basis for the treatment of related disorders in the future.

## Introduction

Hemorphins are 4–10 amino acid-long peptides resulting from the sequential cleavage of β-hemoglobin, with a shared tetrapeptide core comprised of tyrosine-proline-tryptophan-threonine (YPWT). These bioactive peptides were extracted from the adrenal tissue, pituitary gland, hypothalamic tissues, central and peripheral nervous systems, and biological fluids, including plasma and cerebrospinal fluid. They are endogenous cryptides of the atypical opioid peptide family and have therapeutic significance in blood regulation, transient hypotension, analgesia, spatial learning, memory enhancement, and inflammation reduction^1–4^.

The hemorphin sequence is well-conserved among mammals, except camels, which uniquely feature a Q>R variation following the shared YPWT sequence. Recently, this single amino acid disparity in camels has been studied on several targets using in silico and in vitro techniques^5^. The camel forms of these peptides were reported to exhibit a greater affinity for all the protein targets tested. One such target is angiotensin-1 converting enzyme (ACE1) that cleaves angiotensin-I to angiotensin-II, a potent vasoconstrictor hormone in the renin–angiotensin system (RAS)^6^. ACE1 inhibition is a strategy employed for the management of hypertension. Furthermore, the direct action of hemorphins on ACE1 has been reported to induce antihypertensive effects^7^. In general, ACE2, which is the ACE1 homolog and the primary host factor for SARS-CoV-2^8^, opposes the action of ACE1 through the conversion of angiotensin-II to angiotensin-(1-7)^9^. Since the two ACE homologs share roughly 40% catalytic domain sequence identity^10^, hemorphins could potentially bind to and inhibit the function of ACE2.

Ayoub and Vijayan recently reviewed the physiological and pathophysiological significance of hemorphins through the functional and pharmacological targeting of G protein-coupled receptors (GPCRs) and provided further insights into the molecular basis of hemorphin action^11^. Angiotensin-II type 1 receptor (AT1R) is a GPCR and is positively modulated by the activity of LVV-hemorphin-7 (LVV-Hem7; LVVYPWTQRF)^12^.

The ACE1 catalytic site is divided into three subsites: S1, S2, and S1′. The S1 subsite contains the residues Ala354, Glu384, and Tyr523; the S2 subsite carries Gln281, His353, Lys511, His513, and Tyr520; and the S1′ subsite contains Glu162. The zinc ion (Zn^2+^) coordinating motif significant for initiating the ACE1–ligand complex is held by His383, His387, and Glu411 of ACE1^13^. The Lys118, Glu123, Arg124, Trp220, Glu403, and Arg522 residues were identified as the secondary binding site of ACE1, alluding to the flexibility of the ACE1 active region in the face of a wide range of potential ACE1 blockers^14^. Table 1 shows the corresponding active site and Zn^2+^-coordinating residues of ACE2^15^.

**Table 1:** Differences in subsite and Zn^2+^-binding region residues in ACE1 and ACE2 (adapted from Lubbe et al.^15^).

S1		S1′		S2		Zn^2+^-binding region	
ACE1	ACE2	ACE1	ACE2	ACE1	ACE2	ACE1	ACE2
Ser555	Thr347	Glu162	Glu145			His383	His374
Phe512	Phe504	Thr166	Asn149				
His513	His505		Arg273	Gln281		His387	His378
Val518	Tyr510	Cys352	Cys344	Lys511	Leu503		
Arg522	Arg514	His353	His345	Tyr520	Phe512	Glu411	Glu402
		Ala354	Pro346				
		Gln369	Met360			Glu384	Glu375
		Cys370	Cys361				
		Thr372	Lys363			Tyr523	Tyr515
		Val380	Thr371				

As shown in Table 1, the catalytic glutamate and Zn^2+^-coordinating residues are conserved in both ACE homologs (His383, Glu384, His387, and Glu411 of ACE1 corresponding to His374, Glu375, His378, and Glu402 of ACE2) along with seven additional residues, i.e., Glu162, Cys352, His353, Cys370, Phe512, His513, Arg522, and Tyr523, of ACE1 comparable to Glu145, Cys344, His345, Cys361, Phe504, His505, Arg514, and Tyr515 of ACE2. The poorly conserved residues between ACE1 and ACE2 in and surrounding the catalytic pocket are Thr166, Gln281, Ala354, Gln369, Thr372, Val380, Ser355, Lys511, Val518, and Tyr520 of ACE1 and Asn149, Arg273, Pro346, Met360, Lys363, Thr371, Thr347, Leu503, Tyr510, and Phe512 of ACE2^15^.

Subsequently, using in silico techniques and an in vitro ACE1 inhibition assay, we determined that camel hemorphins inhibited ACE1 more effectively than other mammalian homologs^6^. Previously, hemorphins of longer lengths (hemorphin-7 and LVV-hemorphin-7) of camel and noncamel variants were tested on ACE1. The effect of shorter hemorphin sequences, as listed in Table 2, is presented in this study. To elucidate the relevance of the N-terminal sequences, camel and noncamel VV-hemorphin-6 and VV-hemorphin-7 were included. The camel hemorphin variants are specified, whereas the remaining peptides represent the noncamel hemorphin sequences of humans and other mammals, such as rabbits, sheep, bovines, horses, and wild boars.

**Table 2:** A list of the tested hemorphin peptides.

S. no	Name	Short name	Amino acid sequence
1	Hemorphin-4	Hem4	Tyr-Pro-Trp-Thr
2	Hemorphin-5	Hem5	Tyr-Pro-Trp-Thr-Gln
3	Camel hemorphin-5	Camel Hem5	Tyr-Pro-Trp-Thr-Arg
4	Hemorphin-6	Hem6	Tyr-Pro-Trp-Thr-Gln-Arg
5	Camel hemorphin-6	Camel Hem6	Tyr-Pro-Trp-Thr-Arg-Arg
6	Hemorphin-7	Hem7	Tyr-Pro-Trp-Thr-Gln-Arg-Phe
7	Camel hemorphin-7	Camel Hem7	Tyr-Pro-Trp-Thr-Arg-Arg-Phe
8	LVV-hemorphin-4	LVV Hem4	Leu-Val-Val-Tyr-Pro-Trp-Thr
9	LVV-hemorphin-5	LVV Hem5	Leu-Val-Val-Tyr-Pro-Trp-Thr-Gln
10	Camel LVV-hemorphin-5	Camel LVV Hem5	Leu-Val-Val-Tyr-Pro-Trp-Thr-Arg
11	LVV-hemorphin-6	LVV Hem6	Leu-Val-Val-Tyr-Pro-Trp-Thr-Gln-Arg
12	Camel LVV-hemorphin-6	Camel LVV Hem6	Leu-Val-Val-Tyr-Pro-Trp-Thr-Arg-Arg
13	LVV-hemorphin-7	LVV Hem7	Leu-Val-Val-Tyr-Pro-Trp-Thr-Gln-Arg-Phe
14	Camel LVV-hemorphin-7	Camel LVV Hem7	Leu-Val-Val-Tyr-Pro-Trp-Thr-Arg-Arg-Phe
15	VV-hemorphin-6	VV Hem6	Val-Val-Tyr-Pro-Trp-Thr-Gln-Arg
16	Camel VV-hemorphin-6	Camel VV Hem6	Val-Val-Tyr-Pro-Trp-Thr-Arg-Arg
17	VV-hemorphin-7	VV Hem7	Val-Val-Tyr-Pro-Trp-Thr-Gln-Arg-Phe
18	Camel VV-hemorphin-7	Camel VV Hem7	Val-Val-Tyr-Pro-Trp-Thr-Arg-Arg-Phe

Due to the binding and inhibition of ACE1 by hemorphins as well as the sequence and structural homology between ACE1 and ACE2, we hypothesized that hemorphins could exhibit considerable binding to ACE2. Thus, this study aims to offer structural insights into the underlying molecular mechanism of the interaction of hemorphins with ACE2. Furthermore, molecular docking and molecular dynamics (MD) simulations were used to contrast the mode of interaction of hemorphins with ACE1 and ACE2 and estimate the binding energetics, dynamics, and stability of protein–peptide contacts. In vitro absorbance assays were also performed for comparing the inhibition of camel and noncamel hemorphins.

## Results

### In vitro inhibition assay

In vitro inhibition assays were only performed against ACE1, given that no suitable ACE2 kit was compatible with the Glomax Discover Microplate Reader used in this study.

The 18 hemorphin peptides listed in Table 2 were screened for ACE1 inhibitory activity in a single run at different doses. The eight peptides that exhibited the highest ACE1 inhibitory activity were shortlisted and further tested in triplicate (Table 3 and the Appendix in the Supplementary Materials).

**Table 3:** IC_50_ (mean ± SD) of the peptides tested against ACE1.

Peptide	IC_50_ (μM) ± SD
Camel LVVHem6	5.12 ± 0.70
LVVHem6	6.45 ± 0.95
Camel LVVHem5	6.88 ± 1.89
Camel LVVHem7	11.15 ± 2.18
VVHem6	12.69 ± 1.14
LVVHem7	13.07 ± 2.87
LVVHem5	20.12 ± 1.33
Camel Hem7	23.57 ± 3.70

The ACE1 inhibitory potential of camel LVVHem6, LVVHem6, camel LVVHem5, camel LVVHem7, VVHem6, LVVHem7, LVVHem5, camel Hem7 at different doses was evaluated. Table 3 shows the IC_50_ of the top eight hemorphins in terms of ACE1 inhibition.

**Figure 1:**
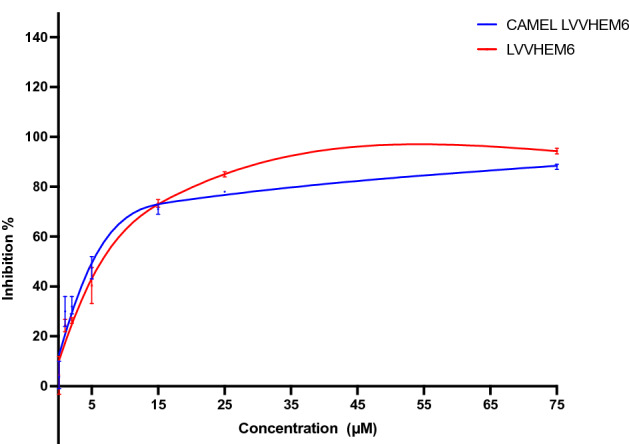
Dose–response curves of camel LVVHem6 and LVVHem6 against ACE1. The data are represented as the mean ± SD of three independent experiments.

The hemorphin peptides exhibited ACE1 inhibitory activity at all doses tested. Camel LVVHem6 (5.12 μM) and LVVHem6 (6.45 μM) reported the lowest IC_50_ values, with reasonable saturation at 75 μM (Fig. 1). The rest of the hemorphin peptides did not reach considerable saturation at the higher doses tested (Fig. S1A). Figure S1B shows the IC_50_ of the top eight peptides in the micromolar range.

### Molecular docking

Given that there was no suitable ACE2 kit accessible for use in in vitro confirmatory experiments, the top performing peptides in terms of ACE1 inhibition, namely camel LVVHem6 and LVVHem6 were considered with ACE2 in in silico docking and MD simulations for the comparative study. In addition, these two peptides appeared on the list of top-scoring peptides in the ACE2 docking (Table 4). The binding conformation of the eight shortlisted peptides that reported the lowest IC_50_ values, i.e., camel LVVHem6, LVVHem6, camel LVVHem5, camel LVVHem7, VVHem6, LVVHem7, LVVHem5, and camel Hem7, was determined through docking and binding free energy calculations.

**Table 4:** Interactions of the best binding pose of the top two peptides with ACE1 (PDB ID: 2XY9) and ACE2 (PDB 1R4L).

Peptide	IC_50_ ± SD against ACE1 (μM)	GlideScore (kcal/mol)	MM-GBSA binding free energy (kcal/mol)	Residues forming hydrogen bonds	Residues forming hydrophobic interactions	Residues forming salt bridge	Residues forming π–π or cation-π contacts
ACE1 (PDB ID: 2XY9)							
Camel LVVHem6	5.12 ± 0.70	−13.66	−139.31	**Glu162**, **Gln281**, **Ala354**, Ala356, Asp377, Asp415, **Lys511**, Ser517, Arg522	Trp59, Tyr135, Leu139, Tyr200, Ile204, Trp220, Trp279, Cys352, **Ala354**, Ala356, Trp357, Tyr360, Cys370, Val379, Val380, Phe391, Tyr394, Pro407, Phe457, Phe512, Val518, Pro519, **Tyr520**, **Tyr523**, Phe527	Asp377, Asp415, **Lys511**	His410
LVVHem6	6.45 ± 0.95	−15.61	−119.56	Asn70, **Gln281**, **His353**, **Ala354**, Ala356, Asp415, **Lys511**	Tyr51, Trp59, Tyr62, Ile88, Ala89, Ala122, Ala125, Val351, **Ala354**, Ala356, Trp357, Tyr360, Val379, Val380, Phe391, Tyr394, Pro407, Phe457, Phe512, Val518, **Tyr520**, **Tyr523**, Phe527	Asp415, **Lys511**	His410
ACE2 (PDB ID: 1R4L)							
Camel LVVHem6		−13.49	−99.50	Asp269, Pro346, Ala348, Asp367, Asp382, Leu391, Asn394, Glu406, Tyr510, Ser511, **Tyr515**	Phe40, Leu95, Ala99, Tyr202, Trp203, Trp271, Phe274, **Pro346**, Ala348, Trp349, Tyr385, Phe390, Leu391, Leu392, Phe504, Tyr510, **Tyr515**	Asp367, Glu406	Phe274, His374
LVVHem6		−12.84	−104.41	**Arg273**, **His345**, **Pro346**, Ala348, Asp350, Leu391, Glu406, Asp509, Tyr510, Arg514, **Tyr515**	Phe40, Trp69, Ala99, Leu120, Val343, **Pro346**, Ala348, Trp349, Leu351, Leu370, Phe390, Leu391, Leu392, Phe504, Tyr510, **Tyr515**	**Arg273**, Glu406	

The residues in bold represent the active site.

Table 4 presents the binding scores and amino acid residues of ACE1 and ACE2 that interact with camel LVVHem6 and LVVHem6. Table S1 reports the binding scores and amino acid residues of ACE1 and ACE2 for LVVHem6, camel LVVHem5, camel LVVHem7, VVHem6, LVVHem7, LVVHem5, and camel Hem7.

**Figure 2:**
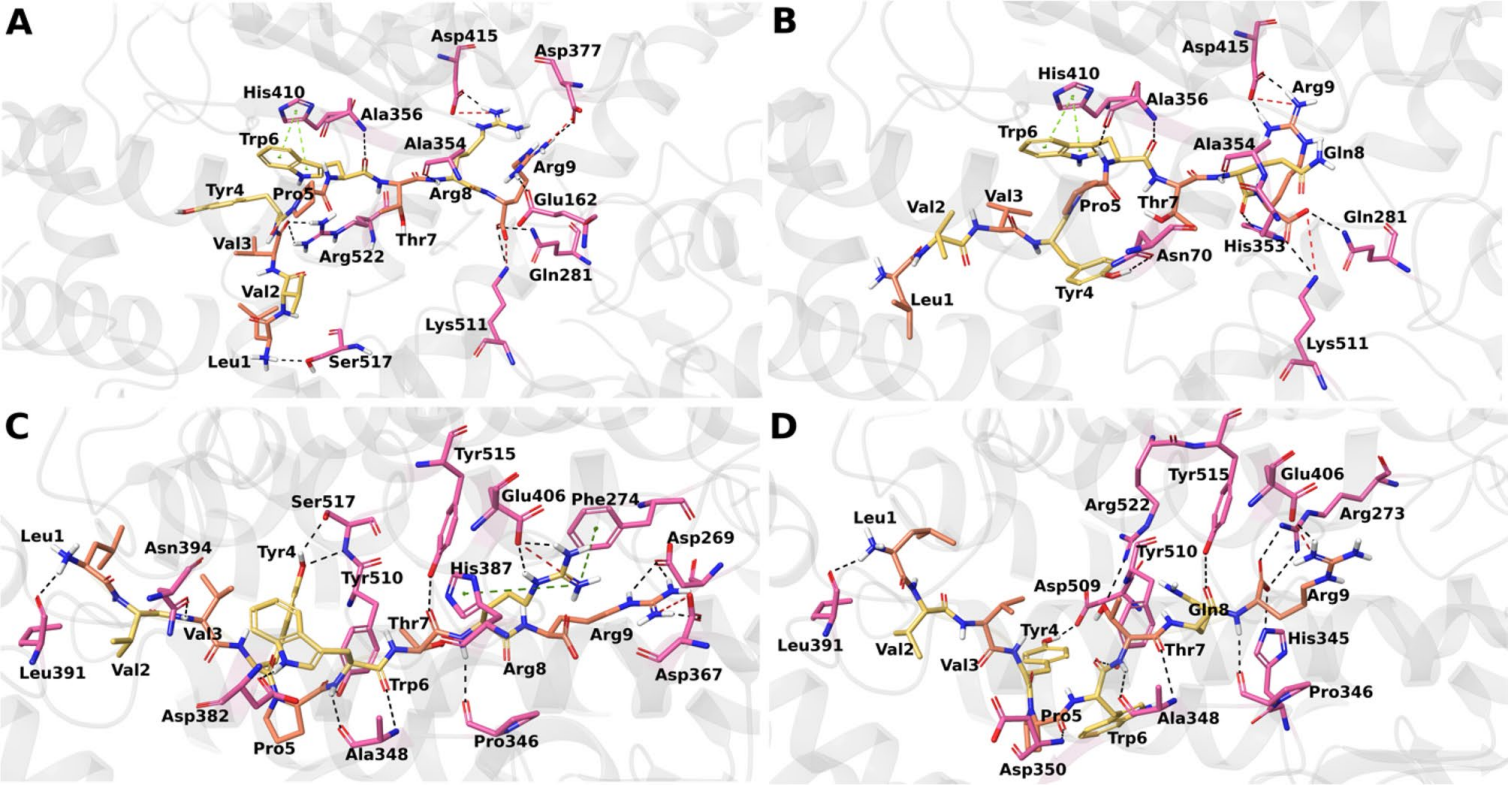
Docked confirmation and hydrogen bond interactions of the hemorphin peptides bound to ACE1 and ACE2. (**A**) Camel LVVHem6 with ACE1 (**B**) LVVHem6 with ACE1 (**C**) Camel LVVHem6 with ACE2 (**D**) LVVHem6 with ACE2. ACE1 and ACE2 are shown in gray cartoon ribbons and their interacting residues are illustrated in a pink stick display; the docked ligand is shown in yellow and orange stick representation, hydrogen bonds are shown as black dashed lines, salt bridges are shown in red, π–π stacking depicted by light green dashed lines, and π-cation stacking is shown in dark green dashed lines.

All peptides were docked in at least one of the ACE1 subsites in the catalytic pocket, i.e., S1, S2, and S1′. Camel LVVHem6 and LVVHem7 docked into all three subsites; LVVHem6, camel LVVHem5, camel LVVHem7, and LVVHem5 occupied two subsites; and VVHem6 and camel Hem7 docked in just one subsite. Figure 2 depicts the docked conformation and hydrogen bond interactions of camel LVVHem6 and LVVHem6 bound to ACE1 and ACE2, while Figs. S5 and S6 depict the same information for the remaining peptides.

All peptides exhibited a hydrophobic interaction with Ala354 at S1 and Tyr523 at S2 (Table 4 and Table S1), excluding camel Hem7, the lowest of the top eight, which did not form a polar nor a hydrophobic interaction with Ala354. The top two peptides in ACE1 binding camel LVVHem6 and LVVHem6 formed an additional hydrophobic association with Tyr520 in S2 (Table 4).

For the optimal binding conformation of camel LVVHem6 with ACE1, binding energies of −13.66 kcal/mol and −139.31 kcal/mol were determined using GlideScore (GScore) and molecular mechanics-generalized Born surface area (MM-GBSA), respectively (Table 4). As the most effective peptide for inhibiting ACE1, it formed hydrogen bonds with all three subsites. At the S1 subsite, the variant Arg8 residue of camel LVVHem6 (Fig. 2A) formed a hydrogen bond and a hydrophobic association with Ala354 (Table 4). It exhibited an additional hydrogen bond and salt bridge with Asp415. The following residue Arg9 featured hydrogen bonding with S2 through Gln281 and also occupied the S1′ subsite via interaction with Glu162. Additionally, Arg9 engages in a salt bridge formation and hydrogen bonding with both Asp377 and Lys511 at S2 (Fig. 2A and Table 4). Additionally, the Trp6 residue mediated a hydrogen bond through Ala356 in S1 and formed π–π interactions with His410.

The LVVHem6 noncamel variant reported the highest GScore of −15.61 mol, kcal/mol, and the MM-GBSA binding energy of −119.56 kcal/mol for its best binding pose (Table 4). Gln8 exhibited a hydrogen bond with Ala354 at the S1 subsite and His353 at the S2 (Fig. 2B). The following Arg9 residue engages in hydrogen bonding with S2 subsite residues Gln281 and Lys511, in addition to a salt bridge with Lys511.

In terms of docking the eight peptides against ACE1 with ACE2, all but LVVHem7 made hydrophobic contact with Pro346 and Tyr515 (Table 4), which corresponds to Ala354 and Tyr523 at the S1 subsite of ACE1 (Table S1). This could partly justify the low MM-GBSA score exhibited by LVVHem7.

The optimal binding conformation of camel LVVHem6 with ACE2 had a GScore of −13.49 kcal/mol and an MM-GBSA binding energy of −99.50 kcal/mol (Table 4). Thr7 engages Tyr515 of ACE2, which is equivalent to Tyr523 of ACE1 (Fig. 2C). Additionally, Arg8 was featured in a single hydrogen bond association with Pro346 (ACE1 Ala354), two hydrogen bond interactions, and a salt bridge with Glu406. Furthermore, it displays cation-π contact with Phe274 and His374 (ACE1 His383) at the Zn^2+^-binding motif.

The optimal noncamel LVVHem6 binding pose exhibited a GScore of −12.84 kcal/mol and an MM-GBSA binding energy of −104.41 kcal/mol (Table 4). Terminal arginine mediates almost all contacts at the active site made by LVVHem6, forming two hydrogen bonds and a salt bridge with Arg273 of ACE2 (ACE1 Gln281) (Fig. 2D). Additionally, it shows contact with His345 and Pro346 of ACE2 (ACE1 His353 and Ala354), respectively. The other active site contacts are mediated by Gln8, which engages the conserved Tyr515 region of ACE2 (ACE1 Tyr523).

VVHem6 was positioned differently in the ACE1 catalytic pocket relative to the other peptides, possibly due to the lack of an N-terminal leucine residue. Consequently, this peptide did not secure polar contact at the active site ACE1. Its top-binding pose reported a GScore of −13.50 kcal/mol and an MM-GBSA of −90.80 kcal/mol (Table S1). Notably, camel Hem7 also did not have active site contact with ACE1, although the best binding pose reported a GScore of −13.052 kcal/mol and a binding energy of −104.15 kcal/mol (Table S1). The N-terminal segment did not show contact with ACE1. Instead, Trp6 formed a hydrogen bond with Ala356 and π–π stacking with His387 in proximity to the S1 subsite. This observation suggests the allosteric binding of camel Hem7 to ACE1.

### MD simulations

Desmond was used to evaluate the stability and dynamics of the interactions between hemorphins and ACE1 and ACE2 by performing 100 ns MD simuations ^16^. Extended simulations of 500 ns were performed for camel LVVHem6 and LVVHem6 with ACE1 and ACE2 since these two peptides reported the lowest IC_50_ in ACE1 in vitro assays.

The MD results show that all 16 complexes retained structural stability throughout the 100 ns simulations, as illustrated by the Cα root mean square deviation (RMSD) (Fig. S2) and the root mean square fluctuation (RMSF) plots (Fig. S3) of the Cα atoms of ACE1 and ACE2.

### Secondary structure of the protein

The composition and compactness of the secondary structure of the proteins were also well maintained and well preserved, as shown by the radius of gyration (Rg) of ACE1 and ACE2 and the hemorphin peptides (Fig. S4). The RMSD of the ACE1-hemorphin and ACE2-hemorphin complexes was predominantly less than 2 Å and did not exceed 3 Å (Fig. S2).

**Figure 3:**
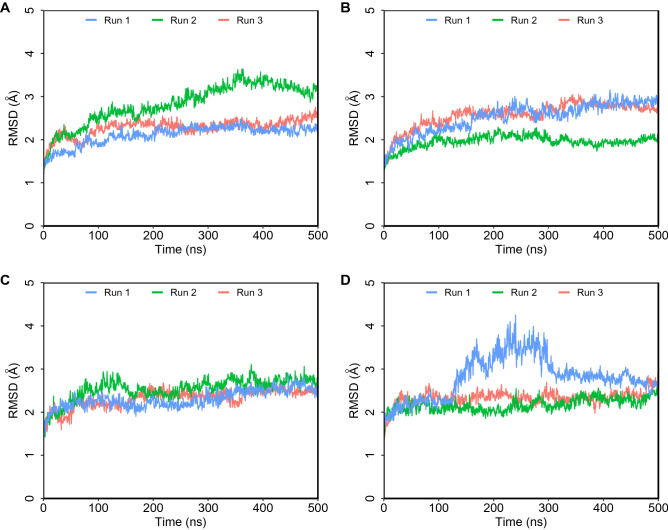
Root mean square standard deviation (RMSD) of protein Cα atoms obtained from 500 ns simulations. (**A**) Camel LVVHem6 with ACE1 (**B**) LVVHem6 with ACE1 (**C**) Camel LVVHem6 with ACE2 (**D**) LVVHem6 with ACE2.

**Figure 4:**
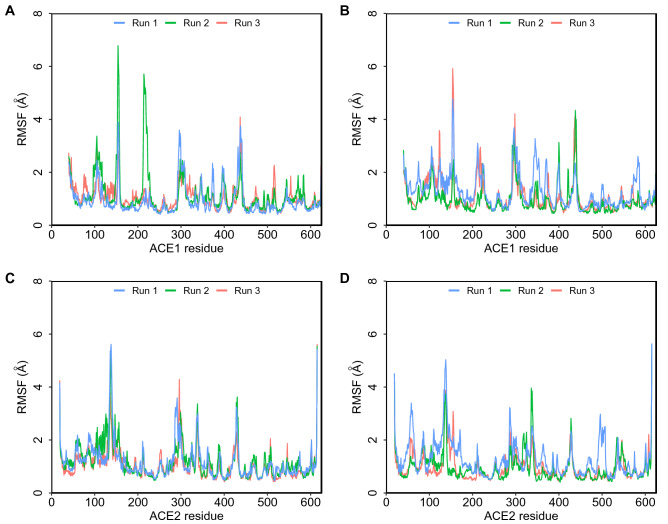
Root mean square fluctuation (RMSF) plots obtained from 500 ns simulations. (**A**) Camel LVVHem6 with ACE1 (**B**) LVVHem6 with ACE1 (**C**) Camel LVVHem6 with ACE2 (**D**) LVVHem6 with ACE2.

**Figure 5:**
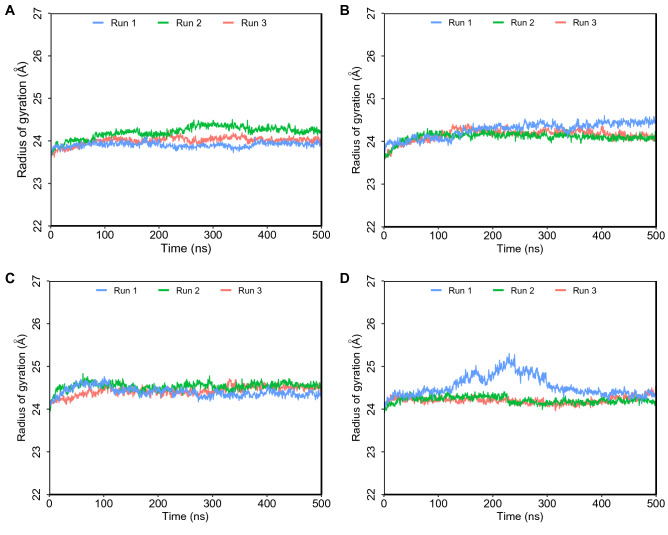
Radius of gyration (Rg) of hemorphin peptides from 500 ns simulations. (**A**) Camel LVVHem6 with ACE1 (**B**) LVVHem6 with ACE1 (**C**) Camel LVVHem6 with ACE2 (**D**) LVVHem6 with ACE2.

The fluctuation depicted by RMSF shows that most ACE1 protein residues show limited fluctuations, in addition to loop regions in some of the complexes (Fig. S3A–H). For ACE2 hemorphin complexes, all structures were equilibrated under an RMSF of 2 Å, with the exception of one terminal residue, which displayed a sharp peak (Fig. S3I–P). This fluctuation potentially corresponds to residues at the boundary between subdomain 1 of ACE2 carrying the catalytic Zn^2+^-binding motif and subdomain 2 at the C terminus with 48% identity to human collectrin^17^. Extended simulations of camel LVVHem6 and LVVHem6 show a similar stable trend in RMSD (Fig. 3), RMSF (Fig. 4), and Rg (Fig. 5).

**Figure 6:**
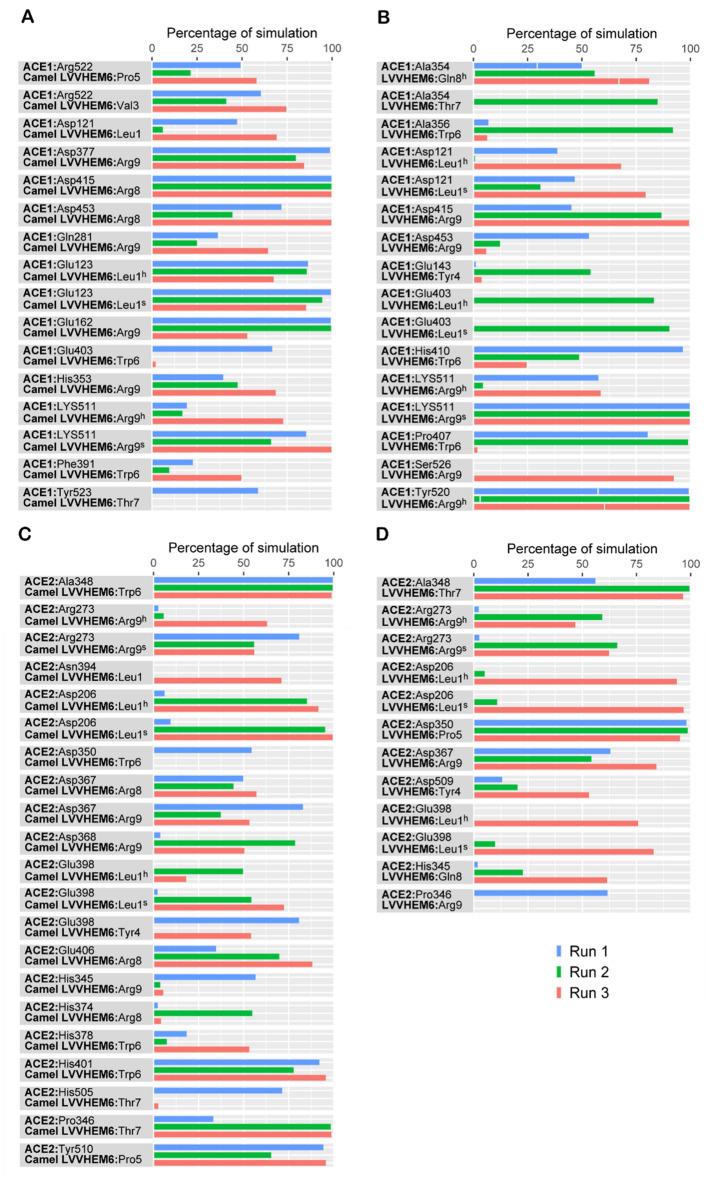
Percentage of contact time during which intermolecular polar contacts were retained between ACE1 and ACE2 and hemorphin peptides in the 500 ns systems. (**A**) Camel LVVHem6 with ACE1 (**B**) LVVHem6 with ACE1 (**C**) Camel LVVHem6 with ACE2 (**D**) LVVHem6 with ACE2. Here, h signifies a hydrogen bond and s signifies a salt bridge.

**Figure 7:**
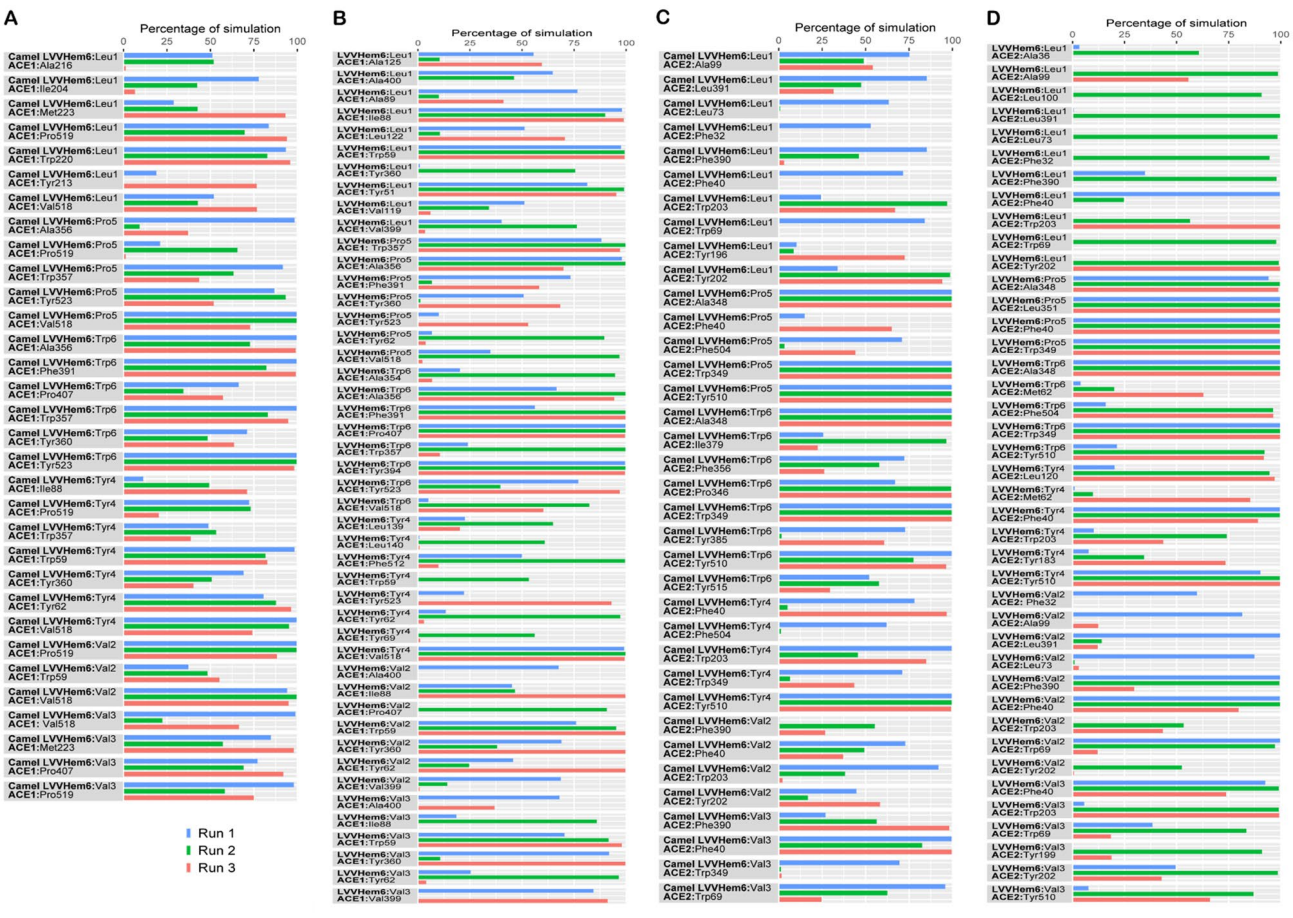
Percentage of contact time during hydrophobic interactions between ACE1 and ACE2 proteins and hemorphin peptides. (**A**) Camel LVVHem6 with ACE1 (**B**) LVVHem6 with ACE1 (**C**) Camel LVVHem6 with ACE2 (**D**) LVVHem6 with ACE2.

The residue-level polar and hydrophobic interactions made by the hemorphin peptides with ACE1 and ACE2 were analyzed for more than 50% of the simulation period of 100 ns (Tables S2–S5) and the extended simulation period of 500 ns (Figs. 6 and 7).

As the peptide reporting the lowest IC_50_ and the best MM-GBSA binding free energy against ACE1, camel LVVHem6 exhibited the highest number of contacts at the ACE1 active site, occupying all three subsites (Tables S2 and S3) through the C-terminus residues Thr7, Arg8 and Arg9. Additionally, Thr7 engaged both His513 and Tyr523 through a hydrogen bond, while Arg8 interacted with Ala354 through a hydrogen bond and Asp415 through a salt bridge for the entire duration of the simulation. The C-terminal Arg9 residue makes a hydrogen bond contact with His353 at S2 and a salt bridge with Glu162 at S1′, in addition to a hydrogen bond and salt bridge contact with Lys511 at S2. Tyr4, Pro5, and Trp6 maintain a consistent hydrophobic interaction with Tyr523 of ACE1 (Table S3). In the 500 ns simulation of camel LVVHem6 with ACE1 (Fig. 6A), the polar interaction between Arg8 and Asp415 was maintained throughout the duration of all three simulation runs, and additional contact was observed with Asp453. The Arg9 residue maintains its contact with Glu162 and Lys511 in all three systems, whereas its interaction with His353 is relatively weaker. Additionally, the terminal arginine exhibits significant contact with Gln281 in one simulation and significant contact with Asp377 in all three systems. Thr7 maintained substantial contact with Tyr523 during the initial simulation. In all three runs, Pro5 and Trp6 mediate hydrophobic associations with Tyr523 at the active site of ACE1 (Fig. 7A).

For the noncamel-LVVHem6 variant, the peptide with the second-lowest IC_50_ and MM-GBSA binding energy, the active site contacts are mediated by the C-terminal Gln8 and Arg9 (Table S2), which occupy all three subsites of ACE1. Gln8 forms a single hydrogen bond with Glu162 at S1′ and His513 at S2, as well as two hydrogen bonds with Ala354 at S1. Hydrogen bonding between Lys511 and Tyr520 and the terminal Arg9 mediates the strong association at S2. A salt bridge creates an additional interaction with Lys511. Trp6 interacts hydrophobically with Ala354 and Tyr523 (Table S3). In extended simulations, Gln8 from LVVHem6 (Fig. 6B) maintains significant contact with Ala354 via hydrogen bonding and a salt bridge in a single system. The terminal Arg9 maintains more interactions with Lys511 and Tyr520. The second simulation features an introduced contact between Thr7 and Ala354. Trp6 maintains greater hydrophobic contact (Fig. 7B) with Tyr523 than Ala354, whereas Tyr4 and Pro5 introduce hydrophobic contact with Tyr523 at the active site within 500 ns, albeit in a single system. While Arg8 of camel LVVHem6 establishes a single interaction with Ala354 at the S1′ subsite, Gln8 of LVVHem6 Gln8 displays three active site interactions with ACE1 and specifically one at each subsite. However, the presence of a substituted arginine in the camel variant increases the total number of active site contacts through Thr7 and Arg9 (Table S2).

In the catalytic pocket, Arg9 of camel LVVHem6 formed a hydrogen bond and salt bridge with Arg273 (ACE1 Gln281), whereas Thr7 formed a hydrogen bond with Pro346 (ACE1 Ala354) (Table S4). The Thr7-Pro346 contact was maintained for nearly the entire duration of the extended simulation in two consecutive runs (Fig. 6C). Additionally, Thr7 has an additional interaction with His505 during the initial run. The terminal Arg9 forms a strong hydrogen bond with Arg273 in the third run and a salt bridge in all three simulations. The hydrophobic associations of camel LVVHem6 at the ACE2 active site were mediated by Tyr4, Pro5, and Trp6, which in total preserved significant contact with Pro346 and Tyr515 of ACE2 (Fig. 7C).

All active site contacts of LVVHem6 with ACE2 (Table S4) are mediated solely by the C-terminal Arg9, which contacts Arg273 (ACE1 Gln281) via a salt bridge. It also features a π-cation interaction with His374 (ACE1 His383), a hydrogen bond with His345 (ACE1 His353) and Pro346 (ACE1 Ala354). Beyond the catalytic region of ACE1, Thr7 mediates a hydrogen bond with Arg514 corresponding to the conserved Arg522 of ACE1. As in the 100 ns system, polar active site associations in the extended simulations are also mediated by terminal arginine through interactions with Arg273 and Pro346 (Fig. 6D). The preceding Gln8 features a significant hydrogen bond interaction with His345 of ACE1 in the third system. Hydrophobic associations at the ACE2 active site (Fig. 7D) are mediated by Val3-Tyr510 and Trp6-Tyr510 in two consecutive simulations, and Tyr4-Tyr510 in all systems.

## Discussion

Recent research has demonstrated that bioactive hemorphin peptides interact with ACE1 and exhibit similar contact characteristics as clinically prescribed ACE1 inhibitors^6,18^. Given that the two ACE homologs share around 40% sequence identity, the hemorphin peptides were tested for their potential to bind to ACE2. This study demonstrates that hemorphins interact with residues that are conserved at the catalytic site of ACE2.

Three disulfide bridges are conserved between the ACE homologs (Cys133–Cys141, Cys344–Cys361, and Cys530–Cys542)^19^. Camel LVVHem6, LVVHem7, and LVVHem7 formed hydrophobic contacts with Cys352 and Cys370 of ACE1, with camel LVVHem7 forming a hydrogen bond with Cys370 (Tables 4 and S1). Two of these peptides, camel LVVHem7, and LVVHem7, mediated the hydrophobic interaction with the Cys344–Cys361 disulfide bridge in ACE2^19^.

Regarding the structural functionality of ACE1 blockers, it has been shown that the high ACE1 inhibitory activity of a peptide is favored by the presence of specific residues at the two termini^20,21^; specifically, a hydrophobic N-terminal portion carrying leucine, valine, isoleucine and glycine^14^, and a C-terminal end that holds several aromatic residues including proline, tyrosine, tryptophan, and phenylalanine; and positively charged residues, particularly arginine, and lysine. Although the N-terminal segment of the hemorphin peptides did not show direct active site engagement except in only one of the 16 complexes, ACE2 Pro346/camel LVVHem7, the inhibition of ACE1 appears to increase with its presence. This indicates that the LVV portion supports the previously described stable binding at the catalytic site^22–25^. Consistent with this report is the two-fold increase in IC_50_ of VVHem6 (12.687 μM) compared to LVVHem6 (6.448 μM) (Table 3).

Furthermore, simulations show that the C-terminal region (Thr7, Arg8/Gln8, Arg9, Phe10) mediates all polar active site interactions with ACE1 (Table S2), while Tyr4 and Pro5 and Trp6 feature the most hydrophobic ones (Table S3). Interactions between hemorphin and ACE2 show a similar pattern (Tables S4 and S5).

ACE1 and hemorphins share a similar interface to that of ACE1 and its clinically relevant inhibitors RXPA380, lisinopril, captopril, and enalaprilat, which interact with Glu162, His353, Ala354, and His383, Glu384, His387, Glu411, Lys511, His513, Tyr520, and Tyr523 of ACE1. Except for His383, ACE1-hemorphin complexes exhibited all these interactions (Tables 4 and S1). Tyr62, Asn66, Lys118, Glu123, Arg124, Trp220, Trp357, Val380, Phe391, Ser355, Glu376, Val379, Val380, Glu403, His410, Phe457, Phe512, Val518, and Arg522 have also been observed to interact directly with ACE1 drugs outside of the catalytic pocket^14^. All of these, except Glu376, were observed in the molecular docking of hemorphin peptides with ACE1 (Tables 4 and S1). All common interactions between ACE1 inhibitors and hemorphin peptides are well preserved in 100 ns simulations, except for those with Glu384 and His387 (Tables S2 and S3). Additionally, the hemorphin-ACE1 interaction also displays interactions that are not observed with those of ACE1 blockers. For example, unlike all the tested hemorphins peptides, none of the ACE1 inhibitors featured interaction with Ala356 close to Ala354 at the active site of ACE1 (Tables 4 and S1).

Furthermore, hemorphin interaction with ACE1 resulted in direct hydrogen bond and salt bridge connections in place of the water-mediated hydrogen bond contact mediated by the ACE1 inhibitors lisinopril and RXPA380 with Asp415^14^ (Tables 4 and S1).

This study’s findings revealed that camel Hem7 (23.57 μM) exhibited the highest IC_50_ with ACE1 among the top eight peptides, and this is consistent with the in silico results. In the docking results, the camel Hem7 only had a single hydrophobic interaction with Tyr523 at the S1 subsite (Table S1). Instead, it uniquely interacted with His387 via an aromatic π-π contact at the Zn^2+^-coordinating motif (Table S1). Similarly, MD simulations revealed two hydrophobic interactions with Ala354 and Tyr523, mediated by its Trp6 residue (Table S3). Its C-terminal residues Arg8 and Phe10 did not exhibit considerable contact in the active region.

Similar to ACE1, the ACE2 inhibitor MLN-4760 mediates direct hydrogen bonds with Arg273, His345, His505, Thr371, and Pro346 of ACE2. It also mediates proximal interactions with Glu145, Asn149, Phe274, Lys363, Asp368, and Met360, as well as a disulfide bond between Cys344 and Cys361 that forms a hydrophobic region at the ACE2 binding site^15,19^. All of these residual contacts, except for Asp368 (Tables 4 and S1), have been observed in the interactions of hemorphins with ACE2. Furthermore, of these contacts, except those with Thr371, Lys363, Met360, and Cys361, are maintained in the 100 ns simulations^19^ (Tables S4 and S5).

Recently, Kesari et al. reported that the purified VFK tripeptide fragment of *Momordica charantia* seeds exhibit a higher ACE1 binding affinity relative to the clinically used lisinopril drug, with both features having hydrogen bond contacts with His353, Glu384, Lys511, and His513^26^. These are among the interactions highlighted between both ACE homologs and the tested hemorphin peptides.

Research has shown a correlation between increased inhibition of ACE1 and positioning of arginine close to the C-terminus^6,27^; due to conformational changes that deeply embed the N-terminal segment in the catalytic pocket, increasing ACE1 inhibition^24,27,28^. In ACE1, camel LVVHem7 and LVVHem6 were observed to form stronger polar contacts than their noncamel counterparts (Table S2). This phenomenon was significantly more pronounced in ACE2 involving the same hemorphin peptides with respect to both polar (Table S4) and hydrophobic interactions with ACE1 (Table S3) and ACE2 (Table S5) in the presence of arginine in the C-terminal segment.

As there was no suitable ACE2 kit available for our in vitro confirmation experiments, the analysis of ACE2 binding relies solely on in silico data presented for comparative evaluation.

As reported by Guy et al.^29^, maintaining a positive charge at position 273 is insufficient for binding to the active site; hence the positive side chain of Arg273 is essential for substrate binding, and the substitution of Arg273Lys renders the enzyme inactive. Furthermore, they demonstrate the importance of His345 and His505, particularly the former, as their substitution significantly decreases the enzyme activity.

Additionally, prior research has revealed that ACE2 peptide ligands^29^ and the C-terminus of the ACE2 inhibitor MLN-4760 engage in a salt bridge with Arg273^30^. In the molecular docking analysis, it was observed that four hemorphin peptides, LVVHem6, VVHem6, LVVHem5, and LVVHem7, form hydrogen bonds with Arg273 (Tables 4 and S1).

Ligand binding at the S2 subsite of ACE1 is involved in positioning peptide substrates for dipeptidyl carboxypeptidase cleavage, and activity since all reported ACE1 native structures feature a ligand docked at this subsite^31–33^. In contrast, none of the residues at the S2 subsite of ACE1 (Gln281, Lys511, and Tyr520) are conserved in ACE2 (Leu503, and Phe512) (Table 1) . Thus, ACE2, unlike ACE1, is a carboxypeptidase that cleaves only a single residue off the C-terminus of its substrates^15^.

Arg273 of ACE2, which is larger than Gln281 of ACE1, is believed to promote steric crowding at the likely S2′ subsite, in addition to stabilizing inhibitors and peptide ligands^19^. This nonconserved residue provides a theoretical explanation for the functional selectivity between ACE1 and ACE2 as peptidyl dipeptidases and carboxypeptidases, respectively, upon elimination of ACE2 S2′. In addition to explaining the inactivity of clinically used ACE1 inhibitors captopril, lisinopril, and enalaprilat against ACE2, such poorly conserved regions could explain the molecular basis of substrate and inhibitor specificity, as well as cleavage activity.

Moreover, nonconserved residues beyond the ACE2 catalytic pocket can influence its inhibitor affinity^15,19^. For example, the ACE2 protein interacts exclusively with side chains that are small to medium in size, such as leucyl and prolyl, at its Tyr510 (ACE1 Val518) and Thr347 (ACE1 Ser355) sites. This is consistent with its documented substrate preferences and demonstrates further specificity. Val518 of ACE1 and Tyr510 of ACE2 form hydrophobic contacts with each of the hemorphin peptides tested (Table 4 and S1). An additional dissimilarity between the two homologous enzymes is the single chloride binding site of ACE2, while ACE1 harbors two; this is seen in their difference in chloride tolerance^29^.

Despite the poorly conserved residues in the S1 subsite, both ACE homologs prefer substrates with a longer sidechain length and hydrophobic residues^19,31,32^.

## Conclusions

In conclusion, all 16 hemorphin peptides docked to ACE1 and ACE2 had inhibitory interactions. Several top-binding peptides displayed well-preserved interactions with critical residues at the interaction interface that correspond to conserved residues in both ACE proteins. The preferential position of arginine near the C-terminus for strong ACE1 binding also applies to ACE2, with an arginine variation introducing additional interactions in camel hemorphins. Evidently, hemorphin peptides with top-free binding energy values interact with indispensable ACE2 residues. The interactions between the hemorphin peptides and the two ACE homologs reflect interactions that contribute to substrate specificity.

Additionally, the difference in residue-level interactions, and implications of poorly conserved regions between the two ACE homologs presented in this study provide insight for future studies aimed at discovering selective and domain-specific inhibitors in the treatment of related diseases. Given the opposing activity of ACE1 and ACE2, the possible dual hemorphin inhibition of these two homologs might disturb the natural balance between the two ACE proteins, which could result in blood pressure dysregulation. Thus, the repurposing of hemorphins in drug development  should consider this important aspect. The modification of hemorphins to accommodate the substrate preferences of the two ACE homologs could be considered.

## Methods

### In vitro ACE inhibition assay

ACE1 inhibition was measured using the absorbance-based colorimetric ACE1 Kit-WST (Dojindo Laboratories, Mashiki-machi, Japan) according to the kit instructions. Custom synthesized hemorphin peptides listed in Table 2 were purchased from Watson Biosciences (Houston, TX, USA). The 18 peptides (Table 2) were initially screened in a single run at concentrations of 0, 10, 50, 100, 200, and 500 µM. The top eight peptides in ACE1 inhibition, i.e., camel LVVHem6, LVVHem6, camel LVVHem5, camel LVVHem7, VVHem6, LVVHem7, LVVHem5, and camel Hem7, were selected for triplicate runs. For camel LVVHem6 and LVVHem6, serial samples of dilutions with concentrations of 0.1, 1, 2, 5, 15, 25, and 75 µM were prepared, along with 5, 10, 25, 35, 50, 75, and 100 µM for camel LVVHem5, camel LVVHem7, VVHem6, LVVHem7, LVVHem5, and camel Hem7. In a 96-well microplate, 20 µL of each hemorphin concentration was pipetted into each well. In addition, 20 µL and 40 µL of deionized water were pipetted into blank 1 and blank 2 wells, respectively, and 20 µL of substrate buffer and 20 µL of enzyme working solution were pipetted into each sample well and blank 1 well.

The microplate was incubated at 37 °C for 1 h. After incubation, 200 µL of indicator working solution was pipetted to each well and then incubated for 5 min at room temperature.

### Absorbance

The absorbance was then measured at 450 nm using a Glomax Discover Microplate Reader (Promega, Madison, WI, USA). ACE1 inhibition was measured using the equation given below:

$$Inhibition\, rate \left(\%\right)=\frac{\left(\mathrm{Blank}1 -\mathrm{ Sample}\right)}{\left(\mathrm{Blank}1 -\mathrm{ Blank}2\right)}\times 100$$

where Blank1 (without a sample but with enzyme working solution) and Blank2 (without a sample and no enzyme working solution) are 20 µL and 40 µL deionized water, respectively.

The half-maximal inhibitory concentration (IC_50_) was determined using GraphPad Prism version 9 (GraphPad, San Diego, CA) from a nonlinear regression inhibition plot against the peptide concentration.

The top eight peptides in terms of ACE1 inhibition were then subjected to in silico analysis.

### Protein structure preprocessing

The three-dimensional structure of ACE1 (PDB ID: 2XY9)^14^ and ACE2 (PDB ID: 1R4L)^19^ were downloaded from the Protein Data Bank (PDB) and preprocessed using the Schrödinger Suite 2021-1 Protein Preparation Wizard^34^. Protein preparation entailed proper assignment of bond orders, adjustment of the ionization state, disoriented group orientation, disulfide bond addition, deletion of unwanted water molecules, capping of the terminus amide, partial charge assignment, and addition of missing atoms and side chains. Additionally, hydrogen atoms were added, and pH 7 was set as the protonation state by default. Lastly, the protein structures were optimized and minimized in order to preserve geometric structural stability^35^.

### Active site identification and grid generation

A receptor grid enclosing the active site was generated by defining the active site residues of ACE1 and ACE2. Default parameters and the OPLS 2001 force field were used for this stage, with a van der Waal scaling factor of 1 and a charge cutoff of 0.25. Then, a cubic space was generated around the centroid of the active site residues of ACE1, and ACE2^35^. The C-domain of ACE1, primarily implicated in blood pressure regulation, was used along with the N-terminal peptidase domain of ACE2^15^.

### Peptide docking

Peptide docking was performed to determine the most likely binding orientation of hemorphin peptides with ACE1 and ACE2, analyze the resulting interfacial molecular interactions, and estimate the binding free energy. Standard precision flexible docking was performed for the docking using Schrödinger Glide version 2021-1 with default parameters^36^. Next, using the 3D builder panel, extended conformations of the peptides were generated to facilitate flexible docking with ACE1 and ACE2. The docked poses were ranked in accordance with the GlideScore (GScore) scoring algorithm^37^. Lastly, the top three GScore values of the best-docked poses were shortlisted for further analysis.

### Analysis of docking results and binding free energy calculation

After docking, Schrödinger Maestro (Schrödinger, LLC, New York, USA) was used for visualization and data analysis of the various types of contacts, including hydrogen bonds, salt bridges, π–π and π-cation contacts, and hydrophobic interactions^38^. The binding free energy of the best-docked poses was computed using the molecular mechanics-generalized Born surface area (MM-GBSA) with Schrödinger Prime using the OPLS 2005 force field and the VSGB 2.0 implicit solvent model^38,39^.

### MD simulations

Given that the function of most proteins depends on their dynamics, it is necessary to explore the dynamics of the docked complexes to assess the strength of intermolecular contacts and the stability of the ACE1-hemorphin and ACE2-hemorphin complexes. Thus, MD simulations of the docked complexes were carried out to assess the dynamics and stability of the best binding conformation of the eight peptides shortlisted in the ACE1 in vitro inhibition assay. The MD simulations were run using Desmond with the OPLS 2005 force field^16^.

Sixteen simulation systems were established: ACE1 and ACE2 with each of the top eight hemorphin peptides against ACE1. One hundred nanoseconds (ns) MD simulations were performed in a single run for each protein–hemorphin complex. Extended simulations of 500 ns were performed in triplicate for camel LVVHem6 and LVVHem6 in complex with ACE1 and ACE2 since these two peptides exhibited the lowest IC_50_ values in the in vitro inhibition assay (Table 3). A single point charge water model was used for system solvation in an orthorhombic box of water molecules, with a buffer distance of 10 Å^40^. Subsequently, the simulation model was neutralized with the required number of counterions, and the salt concentration was set at 0.15 M NaCl. Before running the MD simulations, all systems were subjected to the steepest descent minimization and Desmond's default eight-stage relaxation protocol^41^. The Nose–Hoover thermostat and the isotropic Martyna–Tobias–Klein barostat were used to maintain a temperature of 300 K and a pressure of 1 atm, respectively^42,43^.

Long- and short-range coulombic associations were evaluated with a cutoff of 9.0 Å using the respective smooth particle mesh Ewald (PME) method and short-range approach^44^. A time-reversible reference system propagator algorithm (RESPA) integrator was used with an inner time step of 2.0 fs and an outer time step of 6.0 fs^45^. Data were extracted to simulation trajectories every 100 ps. Finally, the protein–ligand contacts, RMSF, and RMSD of the complexes were calculated from the trajectories and plotted using R version 3.6.3.

## Supplementary Information

Supplementary Information.

## Data Availability

The datasets generated during and/or analyzed during the current study are available from the corresponding author on reasonable request.
