# Supplementary material for: Interaction of hemorphins with ACE homologs
Source: Sci Rep. 2023 Mar 6;13:3743. doi: 10.1038/s41598-023-30771-0 (PMC9987361; doi:10.1038/s41598-023-30771-0)
Supplement: Supplementary file 1 — Supplementary Information. [file 41598_2023_30771_MOESM1_ESM.pdf]

Interaction of hemorphins with ACE homologs

Amie Jobe[1], Priya Antony[1], Suhib Altabbal[1], Yusra Al Dhaheri[1], and Ranjit Vijayan[1,2,3,*]

[1] Department of Biology, College of Science, United Arab Emirates University, PO Box 15551, Al Ain, United Arab Emirates.

[2] The Big Data Analytics Center, United Arab Emirates University, PO Box 15551, Al Ain, United Arab Emirates.

[3] Zayed Center for Health Sciences, United Arab Emirates University, PO Box 17666, Al Ain, United Arab Emirates.

* Correspondence: ranjit.v@uaeu.ac.ae

Supplementary Information

A

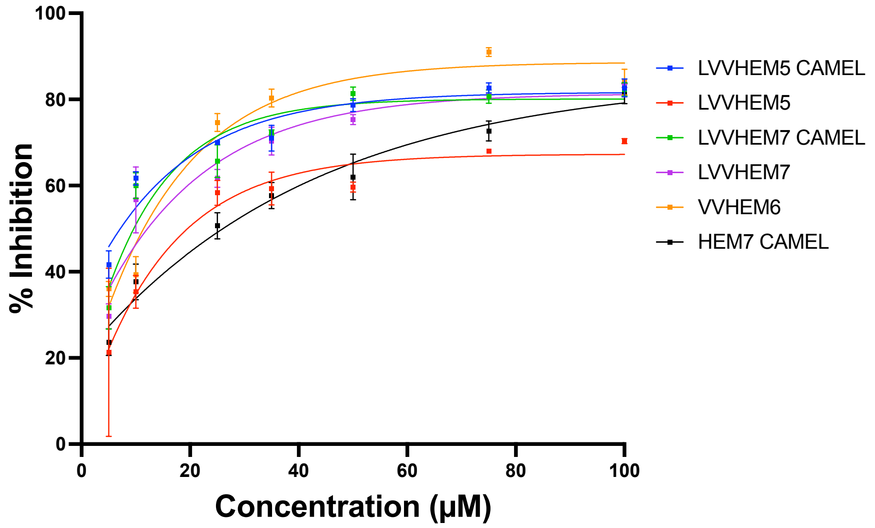

B

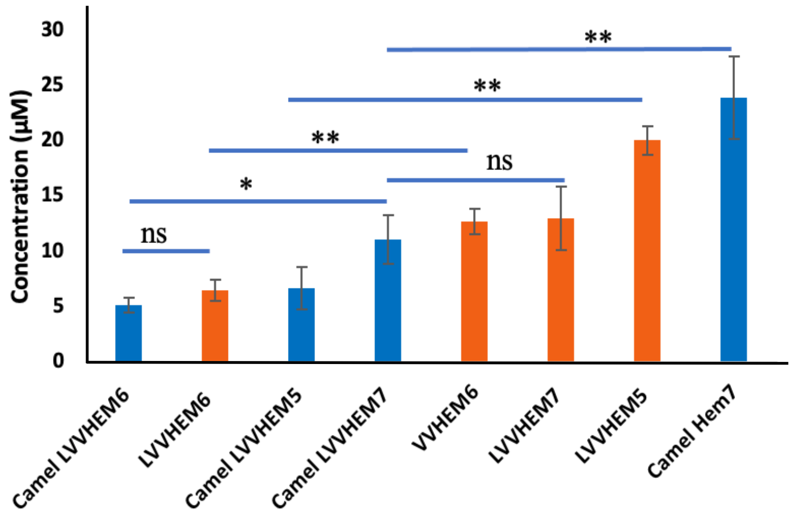

Figure S1: Dose-response curves of the top-eight hemorphin peptides against ACE1. A. Dose-response curves of camel LVVHem5, LVVHem5, camel LVVHem7, LVVHem7, VVHem6, and camel Hem7. Data are presented as mean ± Standard Error of Mean of three independent experiments. B. IC_{50} of the eight peptides expressed in micro molar (μM) units. Data are represented as mean ± SD of the three independent experiments. Statistical significance between hemorphins compared in this study are shown. *** p < 0.001, ** p < 0.01, * p < 0.05, and ns p > 0.05.

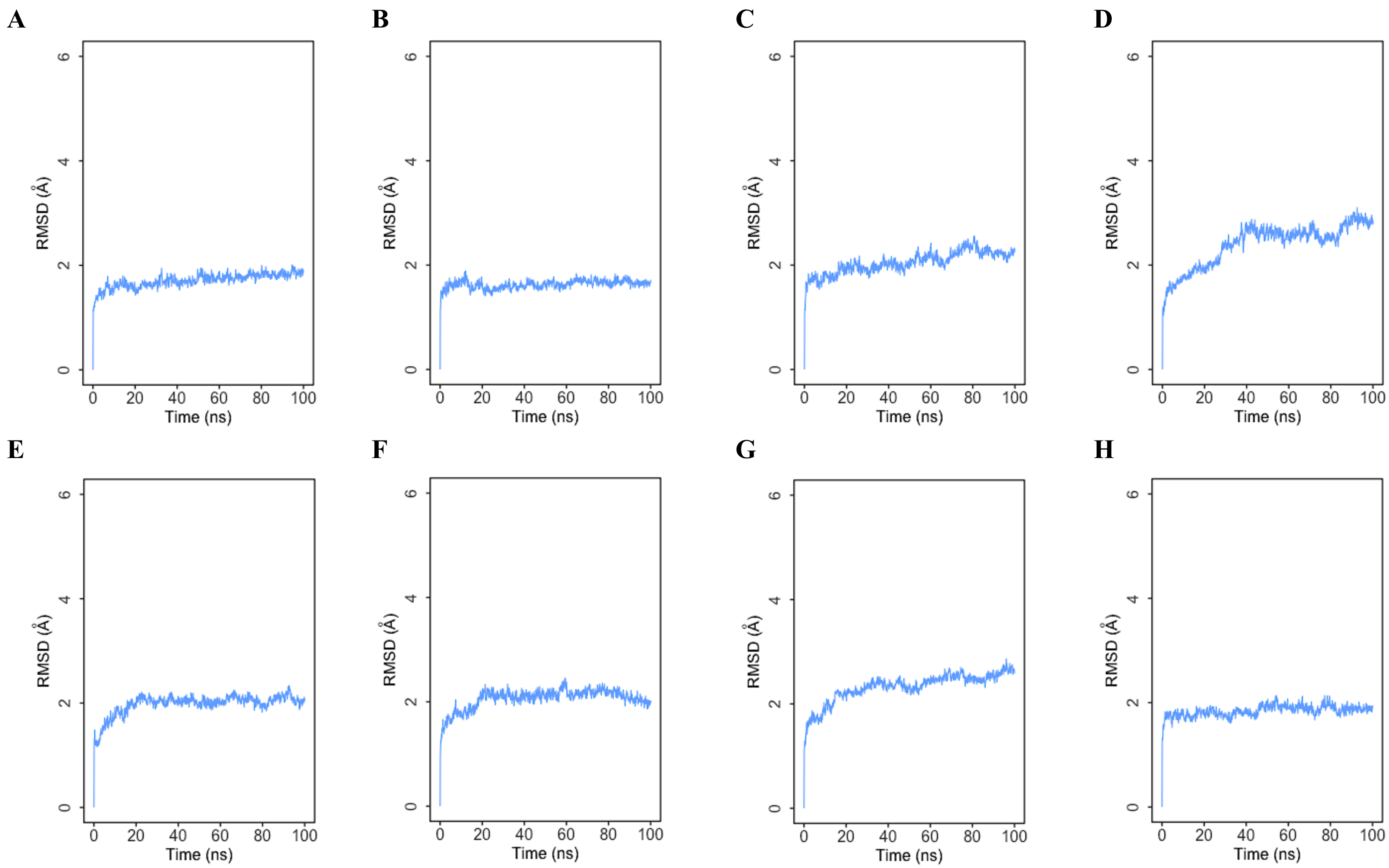

Figure S2: Root mean square standard deviation (RMSD) of protein Cα atoms obtained from the 100ns simulation of the tested hemorphin peptides bound to ACE1 and ACE2. (A) Simulation of camel LVVHem6 with ACE1 (B) Simulation of LVVHem6 with ACE1 (C) Simulation of camel LVVHem5 with ACE1 (D) Simulation of camel LVVHem7 with ACE1 (E) Simulation of VVHem6 with ACE1 (F) Simulation of LVVHem7 with ACE1 (G) Simulation of LVVHem5 with ACE1 (H) Simulation of camel Hem7 with ACE1.

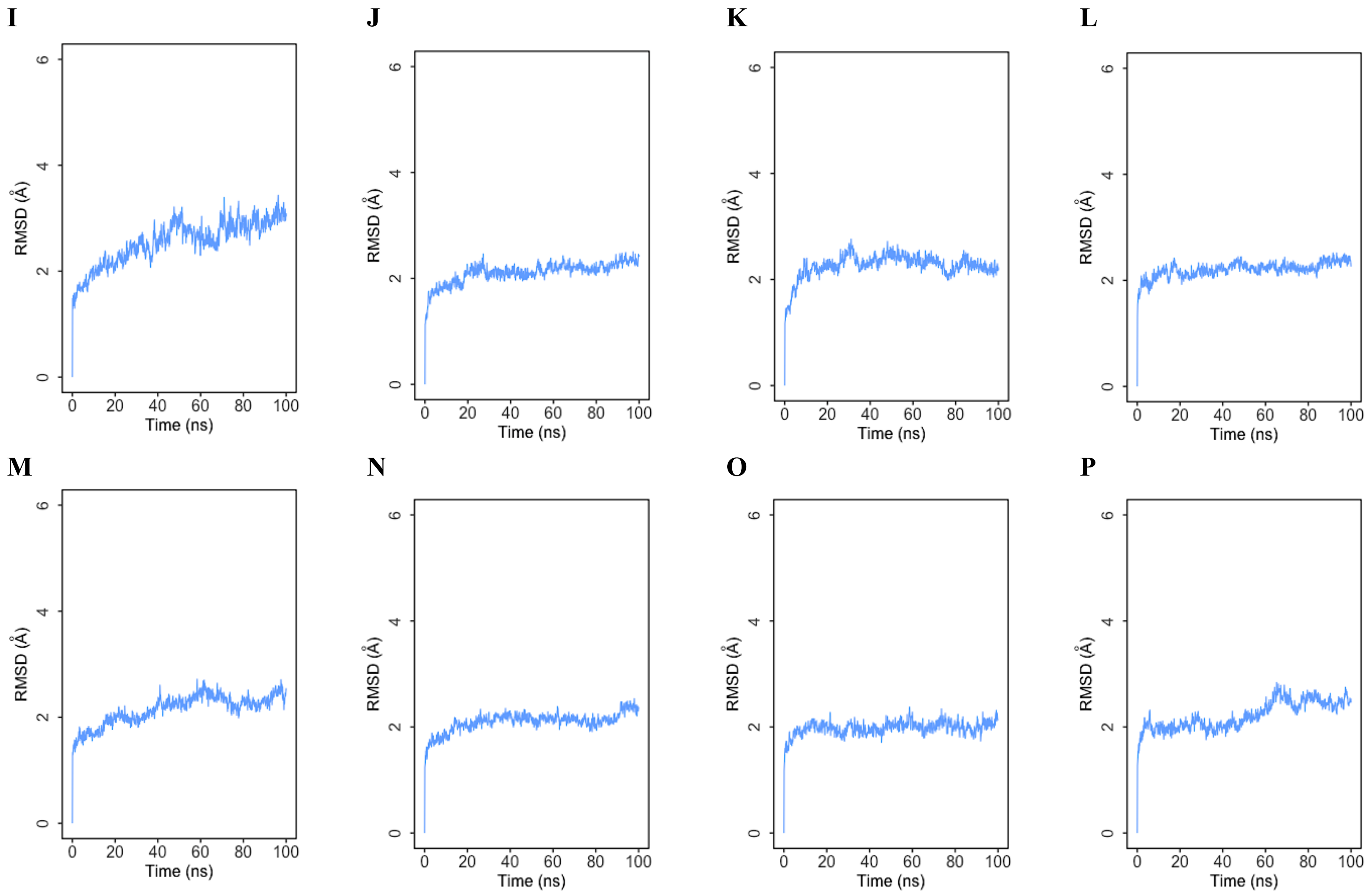

Figure S2: Root mean square standard deviation (RMSD) of protein Cα atoms obtained from the 100ns simulation of the tested hemorphin peptides bound to ACE1 and ACE2. (I) Simulation of camel LVVHem6 with ACE2 (J) Simulation of LVVHem6 with ACE2 (K) Simulation of camel LVVHem5 with ACE2 (L) Simulation of camel LVVHem7 with ACE2 (M) Simulation of VVHem6 with ACE2 (N) Simulation of LVVHem7 with ACE2 (O) Simulation of LVVHem5 with ACE2 (P) Simulation of camel Hem7 with ACE2.

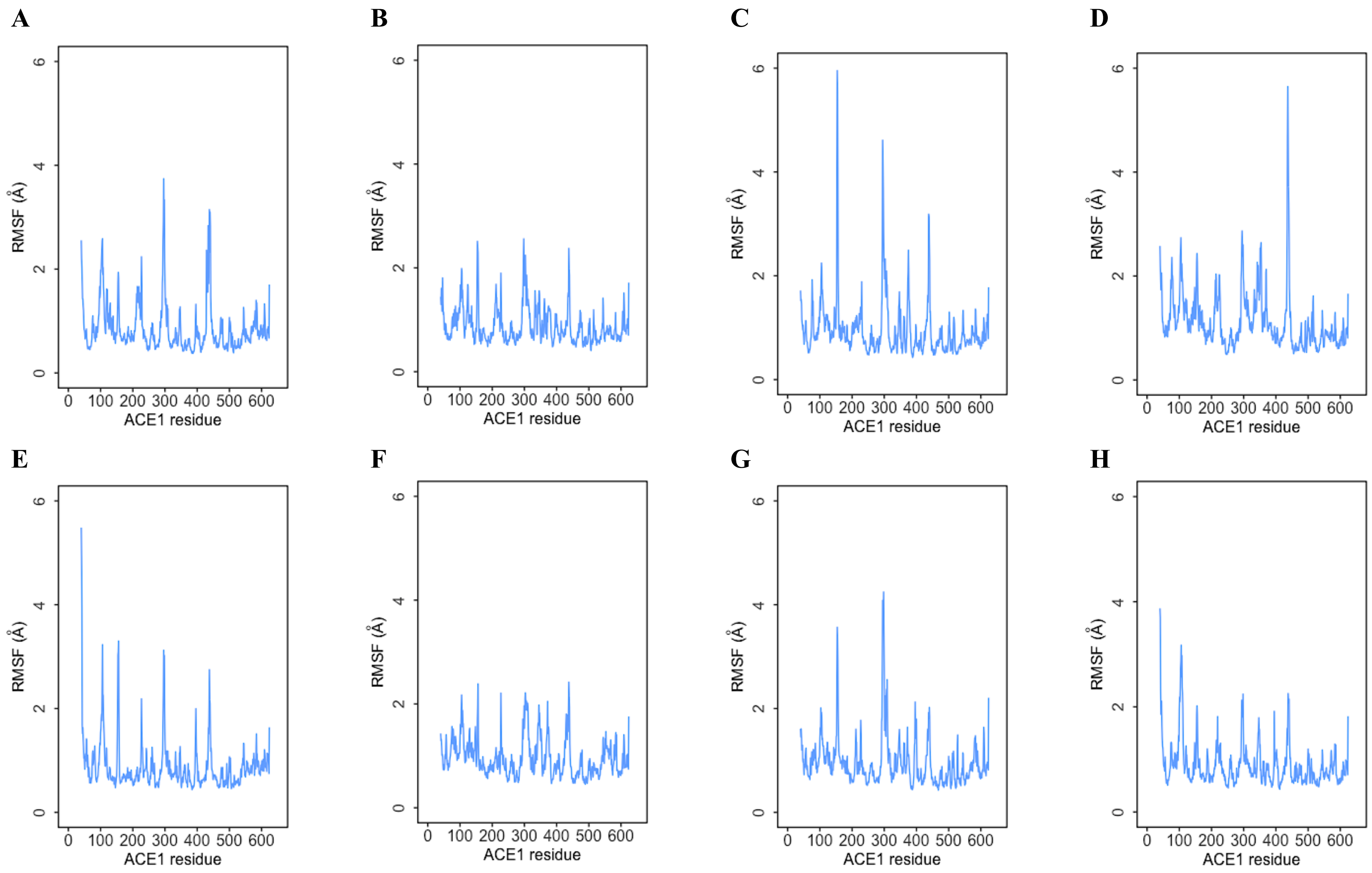

Figure S3: Root mean square fluctuation (RMSF) plots obtained from the 100 ns simulations of the tested hemorphin peptides with ACE1 and ACE2. (A) Simulation of camel LVVHem6 with ACE1 (B) Simulation of LVVHem6 with ACE1 (C) Simulation of camel LVVHem5 with ACE1 (D) Simulation of camel LVVHem7 with ACE1 (E) Simulation of VVHem6 with ACE1 (F) Simulation of LVVHem7 with ACE1 (G) Simulation of LVVHem5 with ACE1 (H) Simulation of camel Hem7 with ACE1.

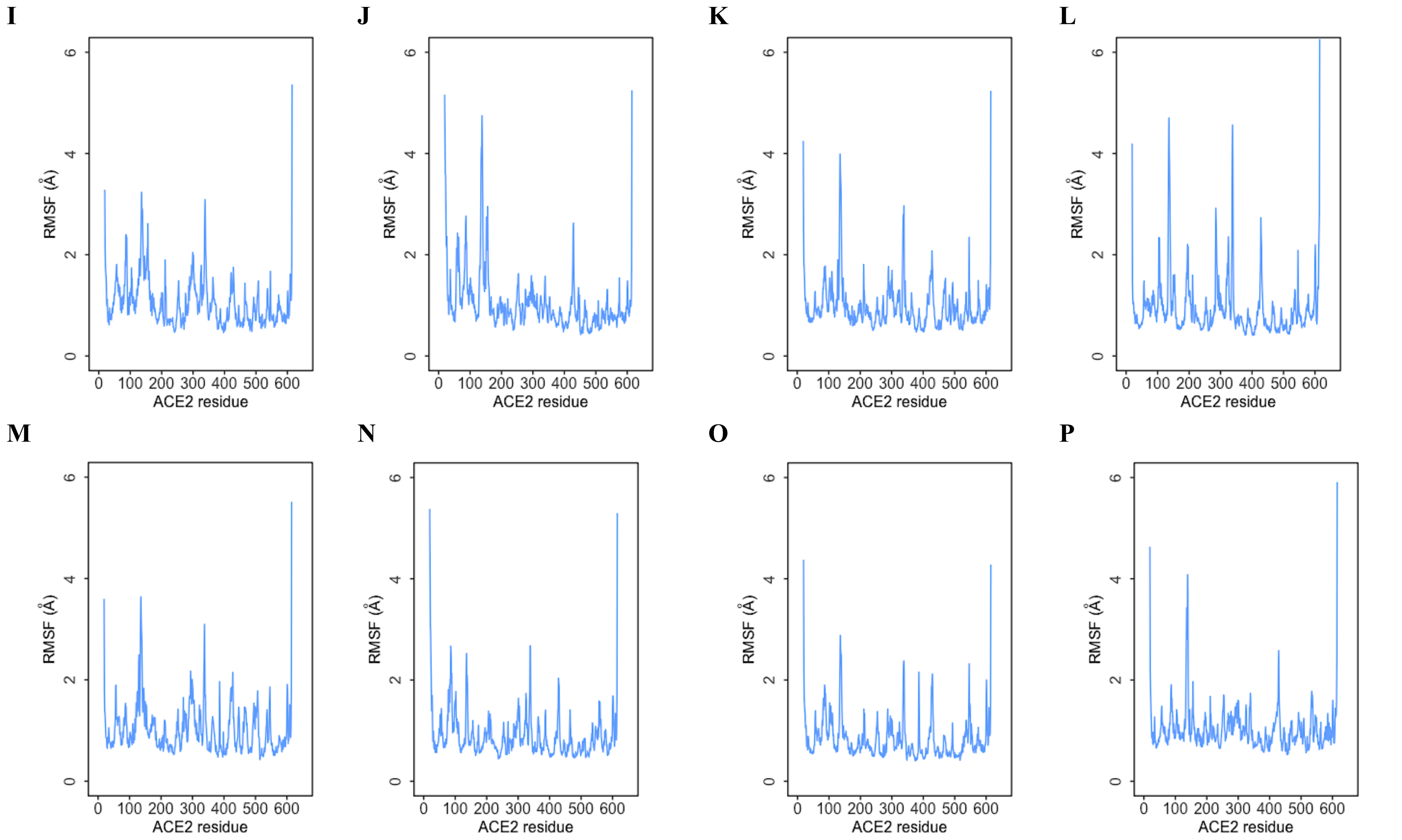

Figure S3: Root mean square fluctuation (RMSF) plots obtained from the 100 ns simulations of the tested hemorphin peptides with ACE1 and ACE2. (I) Simulation of camel LVVHem6 with ACE2 (J) Simulation of LVVHem6 with ACE2 (K) Simulation of camel LVVHem5 with ACE2 (L) Simulation of camel LVVHem7 with ACE2 (M) Simulation of VVHem6 with ACE2 (N) Simulation of LVVHem7 with ACE2 (O) Simulation of LVVHem5 with ACE2 (P) Simulation of camel Hem7 with ACE2.

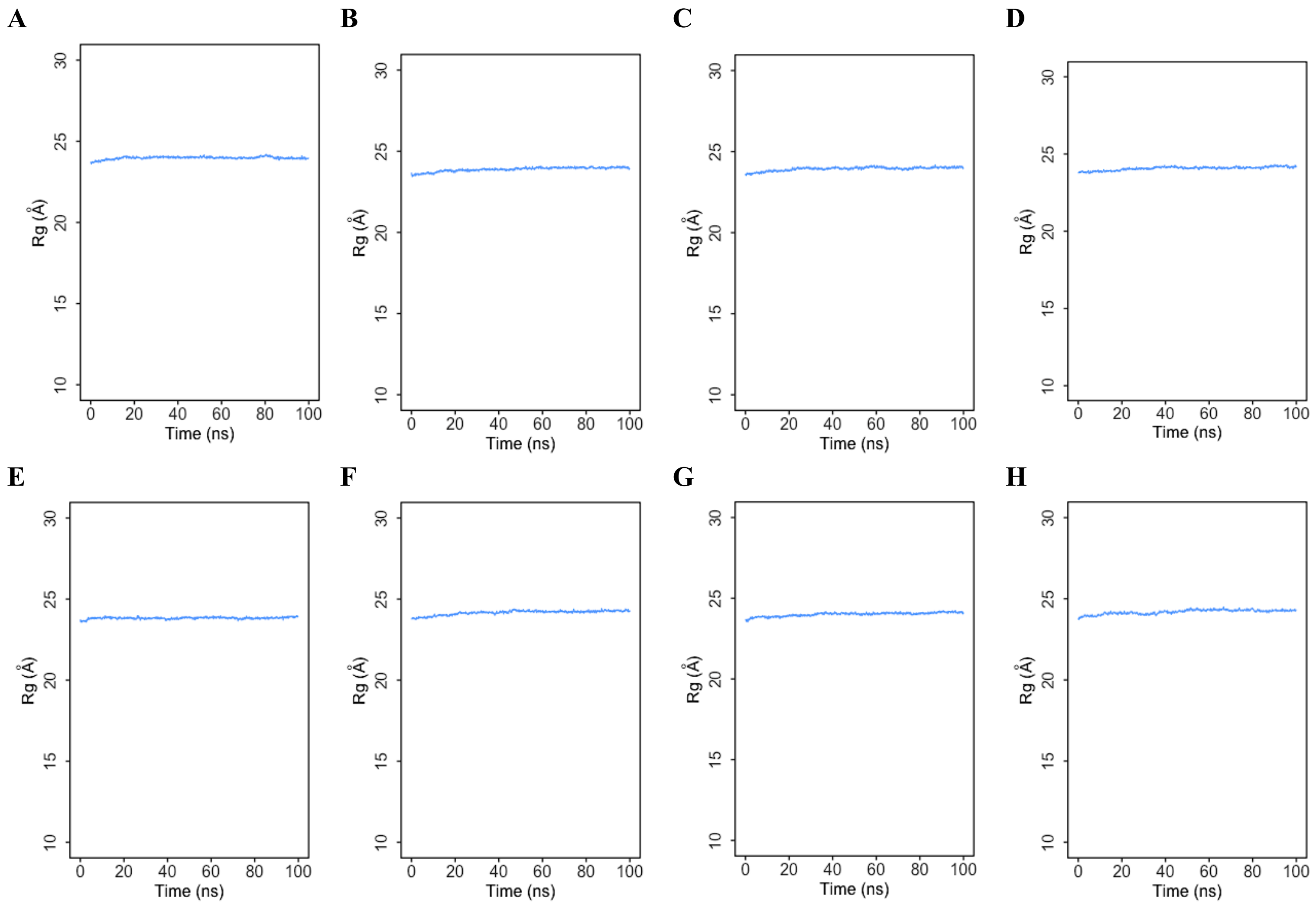

Figure S4: Radius of gyration (Rg) of hemorphin peptides from the 100ns simulations of hemorphins bound to ACE1 and ACE2. (A) Rg of camel LVVHem6 with ACE1 and ACE2. (B) Rg of LVVHem6 with ACE1 (C) Rg of camel LVVHem5 with ACE1 (D) Rg of camel LVVHem7 with ACE1 (E) Rg of VVHem6 with ACE1 (F) Rg of LVVHem7 with ACE1 (G) Rg of LVVHem5 with ACE1 (H) Rg of camel Hem7 with ACE1.

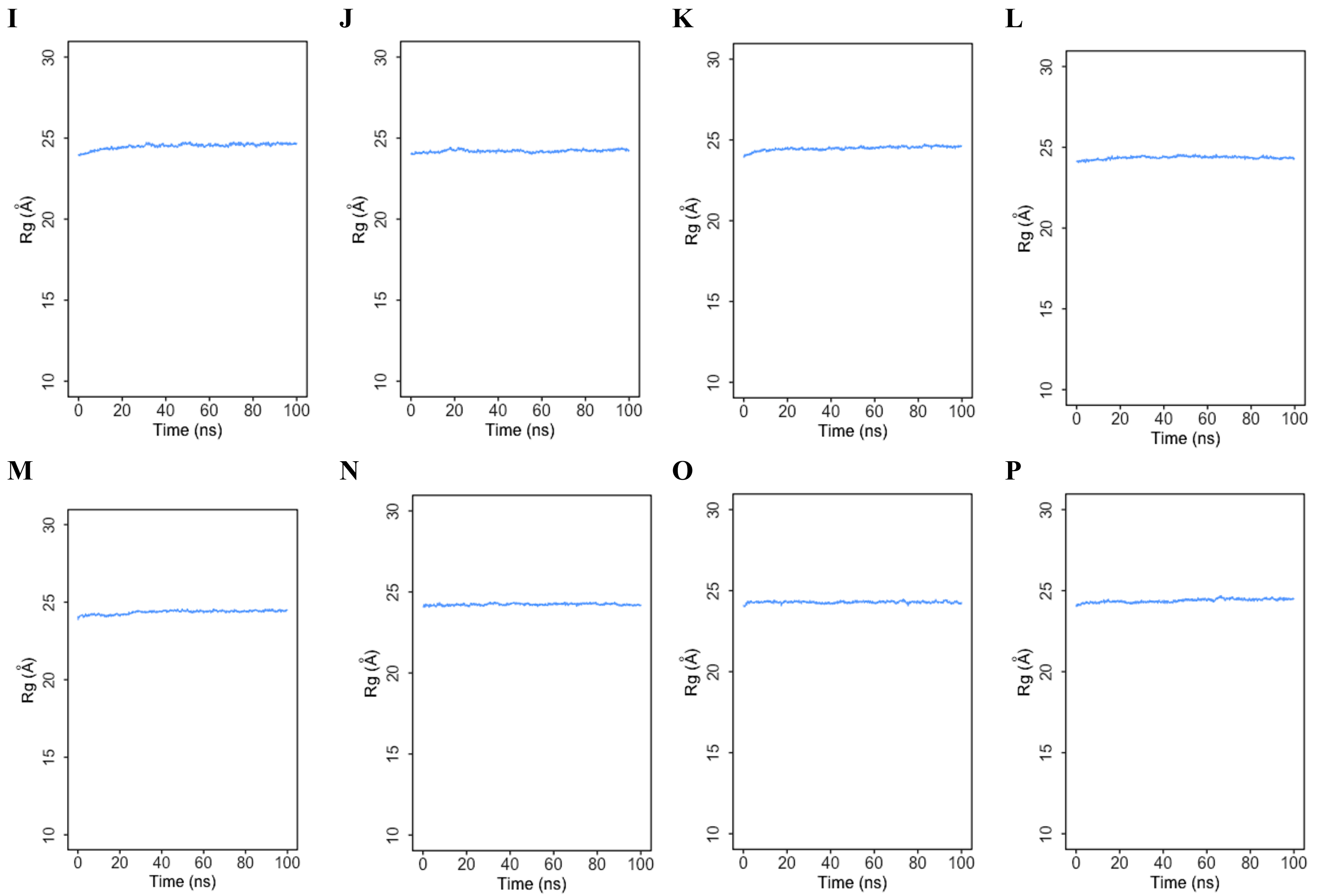

Figure S4: Radius of gyration (Rg) of hemorphin peptides from the 100ns simulations of hemorphins bound to ACE1 and ACE2. (I) Rg of camel LVVHem6 with ACE1 and ACE2. (J) Rg of LVVHem6 with ACE2 (K) Rg of camel LVVHem5 with ACE2 (L) Rg of camel LVVHem7 with ACE2 (M) Rg of VVHem6 with ACE2 (N) Rg of LVVHem7 with ACE2 (O) Rg of LVVHem5 with ACE2 (P) Rg of camel Hem7 with ACE2.

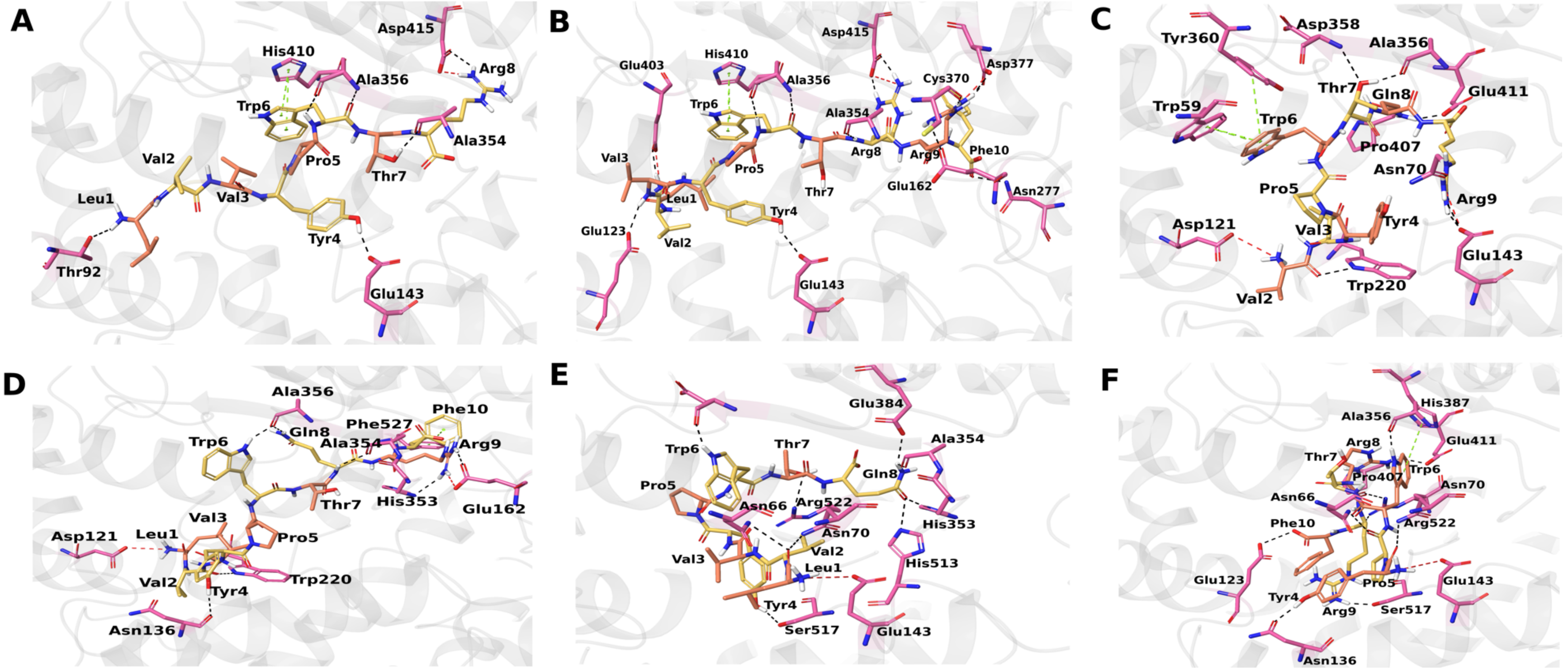

Figure S5. Docked pose and hydrogen bond interactions of the hemorphin peptides bound to ACE1. (A) Camel LVVHem5 with ACE1 (B) camel LVVHem7 with ACE1 (C) VVHem6 with ACE1 (D) LVVHem7 with ACE1 (E) LVVHem5 with ACE1 (F) camel Hem7 with ACE1. ACE1 is displayed in grey cartoon ribbons and its interacting residues are shown in pink stick display; the docked ligand is displayed in yellow and orange stick representation, hydrogen bonds are shown as black dashed lines, salt-bridges are shown in red, π-π stacking depicted by light green dashed lines, and π-cation stacking are shown in dark green dashed lines.

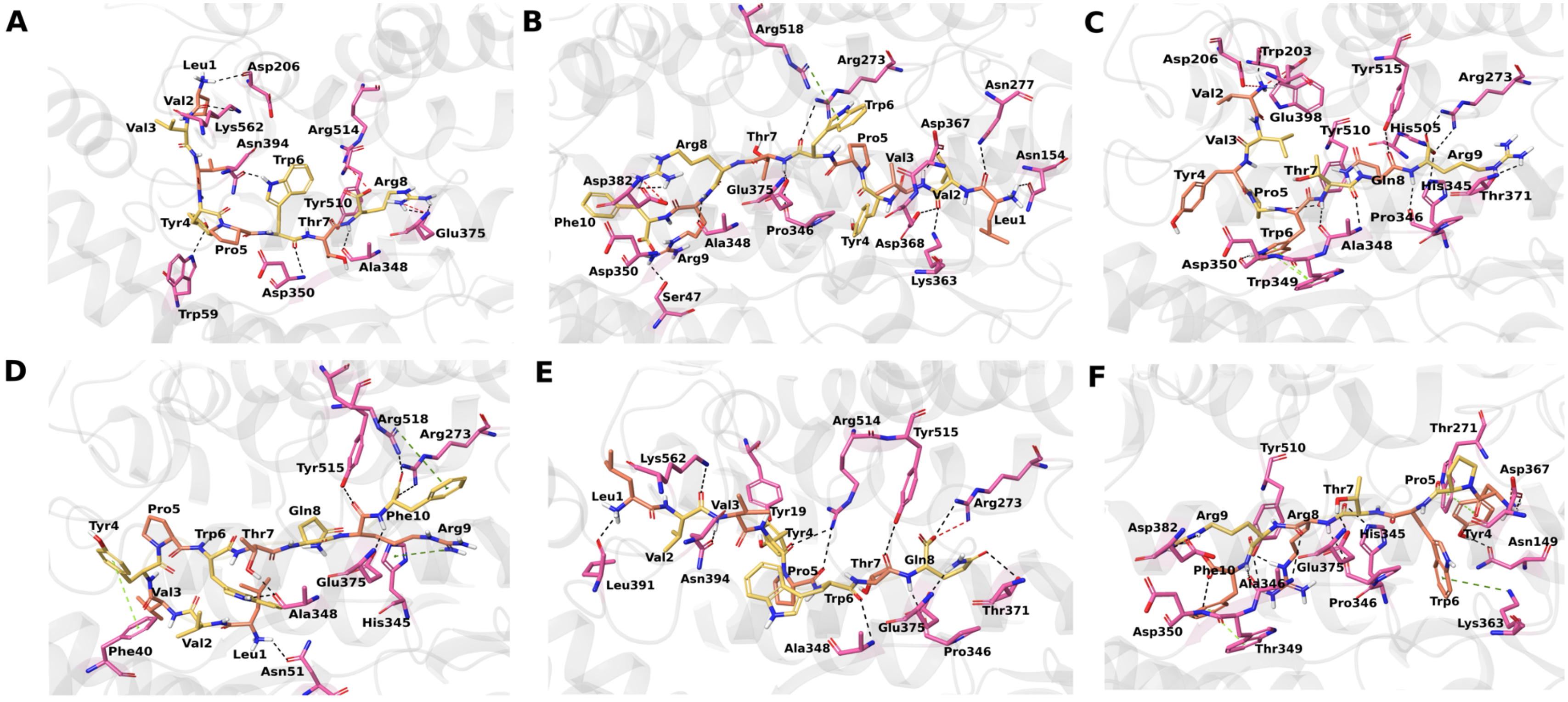

Figure S6. Docked pose and hydrogen bond interactions of the hemorphin peptides bound to ACE2. (A) Camel LVVHem5 with ACE2 (B) camel LVVHem7 with ACE2 (C) VVHem6 with ACE2 (D) LVVHem7 with ACE2 (E) LVVHem5 with ACE2 (F) camel Hem7 with ACE2. ACE2 is displayed in grey cartoon ribbons and its interacting residues are shown in pink stick display; the docked ligand is displayed in yellow and orange stick representation, hydrogen bonds are shown as black dashed lines, salt-bridges are shown in red, π-π stacking depicted by light green dashed lines, and π-cation stacking are shown in dark green dashed lines.

Table S1: Interactions of the best binding pose of the top-three to top-eight peptides with ACE1 (PDB ID: 2XY9) and ACE2 (PDB ID: 1R4L). The residues in bold form the active site.

Peptide	IC_{50} ± SD against ACE1	Glide Score (kcal/mol)	MM-GBSA (kcal/mol)	Residues forming Hydrogen bonds	Residues forming Hydrophobic interactions	Residues forming Salt-bridge	Residues forming π-π or Cation-π contacts
ACE1 – PDB ID: 2XY9							
Camel LVVHem5	6.88 ± 1.89	-13.30	-109.52	Thr92, Glu143, **His353**, **Ala354**, Ala356, Asp415	Tyr51,Trp59, Tyr62, Ile88, Ala89, Leu122, Ala125, Val351, **Ala354**, Ala356, Trp357, Tyr360, Val379, Val380, Phe391, Pro407, Phe512, Val518, **Tyr523**, Phe527	Asp415	His410
Camel LVVHem7	11.15 ±2.18	-16.20	-114.31	Glu123, Glu143, **Glu162**, Asn277, **Ala354**, Ala356, Cys370, Asp377, Glu403, Asp415	Trp59, Tyr62, Ile88, Met223, Trp279, Cys352, **Ala354**, Ala356, Trp357, Tyr360, Cys370, Val379, Val380, Phe391, Pro407, Phe457, Phe512, Val518, Pro519, **Tyr523**, Phe527	Asp377 Glu403 Asp415	His410
VVHem6	12.69 ± 1.14	-13.50	-90.80	Asn70, Glu143, Trp220, Ala356, Asp358, Pro407, Glu411	Trp59, Tyr62, Ala63, Ile88, Leu139, Ile204, Ala207, Ala208, Tyr213, Ala216, Trp220, Met223, Val351, **Ala354** Ala356, Trp357, Tyr360, Phe391, Pro407, Phe512, Val518, Pro519, **Tyr523**	Asp121 Glu143 Zn1628	Trp59 Tyr360
LVVHem7	13.07 ± 2.87	-15.36	-98.54	Asn136, **Glu162**, Trp220, **His353**, **Ala354**, Ala356	Tyr62, Ala63, Leu81, Leu82, Tyr135, Leu139, Leu140, Ile204, Ala207, Ala208, Ala216, Trp220, Met223, Val351, Cys352, **Ala354**, Ala356, Trp357, Cys370, Val379, Val380, Phe391, Pro407, Phe457, Phe512, Val518, Pro519, **Tyr523**, Phe527	**Glu162** Asp121	Phe527
LVVHem5	20.12 ± 1.33	-12.94	-73.52	Asn66, Asn70, **His353**, **Ala354**, Asp358, Glu384 **His513**, Ser517, Arg522	Trp59, Tyr62, Ala63, Tyr69, Leu81, Leu139, Leu140, Ile204, Trp220, Val351, **Ala354**, Ala356, Trp357, Phe359, Tyr360, Phe391, Phe512, Val518, Pro519, **Tyr523**	Glu143 Zn1628	

Camel Hem7	23.57 ± 3.70	-13.05	-104.15	Asn66, Asn70, Glu123, Asn136, Ala356, Pro407 Glu411, Ser517, Arg522	Tyr62, Ala63, Tyr69, Leu81, Tyr135, Leu139, Leu140, Tyr200, Ile204, Trp220, Met223 Val351, Ala356, Trp357, Tyr360, Phe391, Pro407, Val518, Pro519, **Tyr523**	Glu143	His387
ACE2 – PDB ID: 1R4L							
Camel LVVHem5		-12.87	-89.46	Trp69, Asp206, Ala348, Asp350, **Glu375**, Asp394, Tyr510, Lys562	Phe40, Trp69, Leu73, Leu95, Ala99, Tyr196, Tyr202, **Pro346**, Ala348, Trp349, Tyr385, Phe390, Leu391, Leu392 Phe504, Tyr510, **Tyr515**	**Glu375** Arg514 Zn803	
Camel LVVHem7		-15.70	-108.92	Ser47, Asn154, Asn277, **Arg273**, **Pro346**, Ala348, Lys363, Asp367, Ash368,Asp350, **Glu375**, Asp382	Phe40, Tyr127, Leu144, Pro146, Ala153, Trp271, Phe274, Cys344, **Pro346**, Ala348, Trp349, Met360, Cys361, Leu370, Tyr385, Leu503, Phe504, Tyr510, **Tyr515**	Asp382	Arg518
VVHem6		-14.63	-102.25	Trp203, **Arg273**, **His345**, **Pro346**, Ala348, Asp350, Thr371, Glu398, **His505**, Tyr510, **Tyr515**	Phe40, Trp69, Tyr202, Trp203, Phe274, Pro346, Ala348, Trp349, Leu351, Phe390, Phe504, Tyr510, **Tyr515**	Asp206 **Arg273** Glu398	Trp349
LVVHem7		-11.10	-59.09	Asn51, Ala348, **Glu375**, **Tyr515**, Arg518	Phe40, Tyr50, Met62, Trp69, Leu73, Phe274, Pro346, Ala348, Trp349, Leu370, Tyr385, Phe390, Leu391 Phe504, Tyr510, **Tyr515**	**Arg273**	Phe40 **His345** Arg518
LVVHem5		-14.39	-89.63	Tyr199, **Pro346**, Ala348, Thr371, **Glu375**, Leu391, Asn394, Arg514, **Tyr515**, Lys562	Leu95, Ala99, Tyr199, Tyr202, Trp203, Pro346, Ala348, Trp349, Ile379, Tyr385, Leu391, Leu392, Phe504, Tyr510, **Tyr515**	**Arg273**, Zn803	
Camel Hem7		-12.87	-107.81	Asn149, **His345**, **Pro346**, Ala348, Asp350, Asp367, **Glu375**, Asp382, Tyr510	Phe40, Leu144, Ala153, Met270, Trp271, Phe274, Val343, Cys344, **Pro346**, Ala348, Trp349, Leu351, Ala353, Met360, Cys361, Leu370, Tyr385, Phe504, Tyr510, **Tyr515**	Asp367	Trp271 Trp349 Lys363

Table S2. The percentage of contact time during which intermolecular polar contacts were retained between ACE1 and the hemorphin peptides in the 100 ns simulation. The contact is specified when more than one interaction is formed. sb: salt bridge; hb (bb): backbone-backbone hydrogen bond; hb (sb): sidechain-backbone hydrogen bond; hb (bs): backbone-sidechain hydrogen bond.

**Camel LVVHem6**	**Time**	**LVVHem6**	**Time**	**Camel LVVHem5**	**Time**	**Camel LVVHem7**	**Time**
ACE1:ASP377 - ARG9	99.90	ACE1:LYS511 - ARG9 ^{sb}	100	ACE1:ASP415 - ARG8	58.94	ACE1:ASP453 - ARG8	81.42
ACE1:ASP415 - ARG8	100	ACE1:ASP453 - ARG9	56.84	ACE1:ASP453 - ARG8	99.00	ACE1:GLU123 - LEU1 ^{sb}	95.60
ACE1:GLU123 - LEU1 ^{sb}	100	ACE1:GLU123 - LEU1 ^{sb}	91.91	ACE1:GLU123 - LEU1 ^{sb}	75.12	ACE1:GLU162 - ARG9	74.93
ACE1:GLU162 - ARG9	65.13	ACE1:ALA354 - GLN8	98.10	ACE1:LYS511 - ARG8	100	ACE1:ASP121 - LEU1 ^{sb}	83.22
ACE1:LYS511 - ARG9 ^{sb}	100	ACE1:GLU123 - LEU1 $^{hb(bs)}$	80.22	ACE1:HIS410 - TRP6	82.22	ACE1:ASP121 - LEU1 $^{hb(bs)}$	70.73
ACE1:PHE391 - TRP6	89.31	ACE1:GLU411 - THR7	69.53	ACE1:ALA356 - TRP6	67.23	ACE1:GLU123 - LEU1 $^{hb(bs)}$	93.31
ACE1:ALA354 - ARG8	48.15	ACE1:HIS513 - GLN8	69.43	ACE1:GLU123 - LEU1 $^{hb(bs)}$	61.84	ACE1:ASN277 - PHE10	66.83
ACE1:GLU123 - LEU1$^{hb(bs)}$	85.31	ACE1:LYS511 - ARG9 $^{hb(sb)}$	97.70	ACE1:ALA354 - THR7	97.50	ACE1:GLN281 - PHE10	56.14
ACE1:ARG522 - VAL3	49.35	ACE1:TYR520 - ARG9	100	ACE1:TYR523 - ARG8	63.14	ACE1:THR282 - PHE10	60.24
ACE1:LYS511 - ARG9 $^{hb(sb)}$	46.55	ACE1:GLU162 - GLN8	83.42			ACE1:GLU403 - TRP6	53.95
ACE1:TYR523 - THR7	92.61	ACE1:GLU403 - TRP6	50.45			ACE1:ASP415 - ARG8	69.53
ACE1:HIS353 - ARG9	52.25						
ACE1:HIS513 - THR7	88.21						

**VVHem6**	**Time**	**LVVHem7**	**Time**	**LVVHem5**	**Time**	**Camel Hem7**	**Time**
ACE1:ASP121 - VAL2 ^{sb}	100	ACE1:ASP121 - LEU1	45.85	ACE1:GLU143 - LEU1 ^{sb}	99.90	ACE1:GLU403 - ARG8	100
ACE1:GLU123 - VAL2 ^{sb}	100	ACE1:ASP377 - ARG9 ^{sb}	51.25	ACE1:ASN66 - LEU1 $^{hb(bs)}$	99.40	ACE1:GLU143 - TYR4 ^{sb}	100
ACE1:HIS513 - ARG9	51.35	ACE1:GLU123 - LEU1 ^{sb}	99.50	ACE1:GLU143 - LEU1 $^{hb(bs)}$	91.41	ACE1:ARG124 - PHE10	100
ACE1:TRP59 - TRP6	78.42	ACE1:GLU162 - ARG9	99.90	ACE1:ASN66 - LEU1 $^{hb(sb)}$	99.20	ACE1:GLU123 - ARG9	93.21
ACE1:GLU123 - VAL2 $^{hb(bs)}$	99.90	ACE1:LYS511 - PHE10	100	ACE1:TYR360 - PRO5	45.65	ACE1:HIS410 - ARG8	61.84
ACE1:GLU123 - VAL3	99.90	ACE1:SER516 - TYR4	90.51	ACE1:ARG522 - THR7	93.41	ACE1:LYS118 - PHE10	45.25
ACE1:ASP121 - VAL2 $^{hb(bs)}$	98.90	ACE1:SER355 - GLN8	75.92	ACE1:GLU411 - THR7	95.40	ACE1:SER516 - TYR4	62.54
ACE1:SER219 - VAL2	92.61	ACE1:GLU123 - LEU1 $^{hb(bs)}$	89.91	ACE1:HIS353 - GLN8	47.85	ACE1:GLU143 - TYR4 $^{hb(bs)}$	93.21
ACE1:TRP220 - VAL2	92.71	ACE1:TYR135 - VAL2	55.84			ACE1:SER517 - ARG9 $^{hb(sb)}$	53.85
ACE1:GLU411 - GLN8	99.30	ACE1:HIS513 ARG9	98.00			ACE1:ALA356 - TRP3	53.65
ACE1:SER516 - ARG9	73.43	ACE1:TYR520 - PHE10	100			ACE1:ARG124 - PHE10	90.91
		ACE1:HIS353 - THR7	60.24			ACE1:LYS118 - PHE10	48.25
		ACE1:GLU411 - GLN8	93.11			ACE1:ASN66 - PRO5	88.91
		ACE1:ASP377 - ARG9 $^{hb(ss)}$	48.35			ACE1:SER517 - ARG9 $^{hb(ss)}$	44.96
						ACE1:ASN136 - TYR4	72.43

Table S3. The percentage of contact time during which intermolecular hydrophobic contacts were retained between ACE1 and the hemorphin peptides in the 100ns simulation.

**Camel LVVHem6**		**LVVHem6**		**Camel LVVHem5**		**Camel LVVHem7**	
ACE1-LEU1		ACE1-LEU1		ACE1-LEU1		ACE1-LEU1	
ALA216	67.83	LEU122	99.70	ALA125	50.15	LEU122	52.05
ILE204	99.00	MET223	60.04	ILE88	100	MET223	57.64
PHE472	96.70	TRP220	64.14	LEU122	49.35	TYR360	49.95
TRP220	100	TRP59	65.53	TRP59	100	TYR51	51.05
TYR135	90.71	TYR51	58.74	TYR360	63.54	VAL119	54.75
TYR200	95.60	VAL119	89.31	TYR51	55.94	ACE1-VAL2	
VAL518	87.31	ACE1-VAL2		ALA356	99.90	PRO519	96.30
ACE1-VAL2		MET223	58.04	TRP357	100	TRP220	59.14
LEU139	98.90	PRO519	64.94	ACE1-VAL2		VAL518	99.50
PRO519	100	TRP220	61.64	TYR360	100	ACE1-VAL3	
TYR135	59.34	VAL518	56.04	TYR62	67.23	ILE88	49.55
TYR200	87.61	ACE1-VAL3		ACE1-VAL3		TRP59	97.20
TYR62	49.55	LE88	50.35	TYR62	68.93	TYR360	69.43
VAL518	100	LEU139	49.15	VAL518	96.70	TYR62	80.42
ACE1-VAL3		TRP59	67.83	ACE1-TYR4		VAL518	55.04
MET223	73.83	TYR360	56.44	PHE512	100	ACE1-TYR4	
PRO519	100	TYR62	82.42	VAL518	100	PHE512	88.91
TRP220	90.81	ACE1-TYR4		ACE1-TRP6		PRO519	57.34
VAL518	100	PHE512	99.80	ALA354	83.72	VAL518	100
ACE1-TYR4		VAL351	80.22	ALA356	100	ACE1-PRO5	
ALA356	100	VAL518	100	PHE391	100	ALA356	90.51
PHE391	100	ACE1-PRO5		PRO407	100	TRP357	99.70
TRP357	99.90	ALA356	100	TRP357	99.60	ACE1-TRP6	
TYR360	77.72	PHE391	78.32	TYR394	64.64	ALA356	100
TYR394	98.10	TRP357	100			PHE391	100
TYR523	100	VAL518	77.92			PRO407	75.62
ACE1-PRO5		ACE1-TRP6				TRP357	99.40
ALA356	100	ALA354	82.82			TYR360	84.22
TRP357	100	ALA356	100			TYR523	83.82
TYR523	92.41	PHE391	100			ACE1-PHE10	
VAL518	100	PRO407	100			PHE457	66.83
ACE1-TRP6		TRP357	99.70			TRP279	72.43
ALA356	100	TYR394	90.31			VAL379	89.31
PHE391	100	TYR523	80.82			VAL380	92.31
TRP357	99.90						
TYR394	98.10						
TYR523	100						

**VVHem6**		**LVVHem7**		**LVVHem5**		**Camel Hem7**	
ACE1-VAL2		ACE1-LEU1		ACE1-LEU1		ACE1-TYR4	
ALA126	96.20	MET223	75.72	LEU139	100	LEU139	100
ALA207	100	PRO519	61.34	LEU140	99.80	LEU140	100
ALA208	100	TRP220	84.82	LEU81	99.80	LEU81	100
ALA216	100	ACE1-VAL2		PHE512	96.60	LEU82	84.52
ILE204	100	ALA207	97.90	TRP357	99.40	PHE512	86.61
LEU122	99.60	ALA216	59.74	TYR62	100	TRP357	62.74
TRP220	100	ILE204	100	TYR69	99.80	TYR62	100
TYR135	98.80	LEU139	89.51	ACE1-VAL2		TYR69	100
TYR213	100	TRP220	99.90	PHE512	100	VAL518	79.42
ACE1-VAL3		TYR135	100	TRP357	67.43	ACE1-PRO5	
ALA216	40.86	ACE1-VAL3		VAL518	100	LEU139	100
ILE204	96.60	ILE204	54.15	ACE1-VAL3		TYR62	88.21
MET223	100	LEU139	66.93	TRP59	85.91	VAL518	89.11
PRO519	99.70	PRO519	99.90	TYR360	98.40	ACE1-TRP6	
TRP220	100	TRP220	80.62	TYR62	100	ALA354	97.30
TYR135	70.43	TYR135	60.64	VAL518	99.40	ALA356	99.80
ACE1-TYR4		VAL518	100	ACE1-TYR4		PHE391	56.64
LEU139	100	ACE1-TYR4		MET223	83.02	TRP357	100
MET223	49.25	LEU139	100	PRO519	100	TYR360	49.35
PRO519	100	LEU140	100	TRP220	99.90	TYR523	63.94
TRP220	89.41	LEU81	97.50	VAL518	98.50	VAL518	100
TRP357	51.15	PHE512	63.54	ACE1-PRO5		ACE1-PHE10	
TYR200	72.93	TYR62	98.50	ALA400	91.11	ALA400	99.90
VAL518	100	TYR69	74.23	TRP59	52.65	LEU401	96.10
ACE1-TRP6		VAL518	90.81	TYR360	100	MET223	96.20
ALA125	87.61	ACE1-PRO5		TYR394	77.62	PHE570	80.02
ALA400	85.61	LEU139	59.44	VAL399	91.41	TRP59	83.72
ALA89	97.10	PHE512	99.90	ACE1-TRP6		TYR360	92.51
ILE88	99.70	VAL518	100	ALA356	100	VAL399	99.80
TRP357	52.35	ACE1-TRP6		ALA63	95.40		
TRP59	100	ALA356	97.90	PHE359	82.52		
TYR360	100	PHE391	97.90	PHE391	100		
TYR51	98.70	TRP357	100	TRP357	100		
TYR62	92.11	TYR360	53.35	TYR360	99.80		
VAL399	97.00	VAL518	99.60	TYR394	74.93		
		ACE1-PHE10					
		PHE457	100				
		PHE460	50.25				
		PHE527	99.60				

	TYR520	100		
	TYR523	100		

Table S4. The percentage of contact time during which intermolecular polar contacts were retained between ACE2 and the hemorphin peptides residues in the 100 ns simulation. The contact is specified when more than one interaction is formed. sb: salt bridge; hb (bb): backbone-backbone hydrogen bond; hb (sb): sidechain-backbone hydrogen bond; hb (bs): backbone-sidechain hydrogen bond.

**Camel LVVHem6**		**LVVHem6**		**Camel LVVHem5**		**Camel LVVHem7**	
ACE2:ARG273 - ARG9 [sb]	56.34	ACE2:ARG273 - ARG9	74.93	ACE2:GLU375 - ARG8	99.80	ACE2:ASP382 - ARG8	84.92
ACE2:ASP206 - LEU1 [sb]	99.90	ACE2:ASP206 - LEU1[sb]	95.70	ACE2:ARG514 - ARG8 [sb]	98.50	ACE2:HIS374 - TRP6	94.01
ACE2:ASP367 - ARG8	85.91	ACE2:GLU406 - ARG9	95.30	ACE2:ARG514 - TRP6	98.60	ACE2:PHE274 - TRP6	56.64
ACE2:ASP367 - ARG9	66.03	ACE2:HIS374 - ARG9	72.53	ACE2:PHE390 - TYR4	49.55	ACE2:ALA348 - ARG8 [hb(bb)]	99.10
ACE2:GLU398 - LEU1 [sb]	88.91	ACE2:PRO346 - ARG9	91.41	ACE2:TYR196 - LEU1	48.35	ACE2:ASN149 - VAL2	99.10
ACE2:HIS401 - TRP6	86.61	ACE2:ALA348 - THR7	89.51	ACE2:ASP206 - VAL3	97.80	ACE2:ASN277 - LEU1	80.72
ACE2:ALA348 - TRP6	98.20	ACE2:ASP206 - LEU1 [hb(bs)]	72.63	ACE2:ALA348 - THR7	94.71	ACE2:ALA348 - ARG8 [hb(sb)]	63.64
ACE2:ASN394 – LEU1 [hb]	90.31	ACE2:TYR510 - TRP6	57.04	ACE2:ALA99 - TYR4	89.81	ACE2:TYR510 - THR7	46.15
ACE2:ASP206 - LEU1	94.61	ACE2:HIS345 - ARG9	47.65	ACE2:ARG514 - ARG8 [hb(sb)]	96.80	ACE2:GLU406 - TRP6	99.80
ACE2:GLU398 - LEU1 [hb]	88.91	ACE2:ARG514 - THR7	53.05	ACE2:ARG514 - THR7	99.90		
ACE2:PRO346 - THR7	99.20			ACE2:LYS562 - VAL3	96.90		
ACE2:TYR510 - PRO5	84.22			ACE2:PRO346 - ARG8	83.02		
ACE2:ARG273 - ARG9 [hb]	77.62			ACE2:GLU398 - TRP6	90.41		
ACE2:ASN394 - LEU1 [hb]	51.35						
**VVHem6**		**LVVHem7**		**LVVHem5**		**Camel Hem7**	
ACE2:ARG273 - ARG9 [sb]	100	ACE2:ARG273 - :PHE10	100	ACE2:ARG518 - GLN8	86.21	ACE2:ASP367 - TYR4	80.62
ACE2:ASP206 - VAL2 [sb]	100	ACE2:ARG518 - PHE10	48.65	ACE2:PHE390 - LEU1	76.92	ACE2:ASP382 - ARG9	51.15
ACE2:GLU398 - VAL2 [sb]	100	ACE2:GLU145 - ARG9	95.80	ACE2:PHE40 - LEU1	69.33	ACE2:TRP649 - PHE10	90.71
ACE2:ASP367 - ARG9 [sb]	65.23	ACE2:SER124 - LEU1	46.85	ACE2:HIS401 - TRP6	99.50	ACE2:HIS345 - TRP6	50.55
ACE2:ASP206 - VAL2 [hb]	84.22	ACE2:ALA348 - THR7	75.32	ACE2:PHE390 - LEU1	69.63	ACE2:ALA348 - PHE10	98.80
ACE2:GLU398 - VAL2 [hb]	99.90	ACE2:ARG273 - PHE10	100	ACE2:PRO346 - GLN8	53.65	ACE2:ASP367 - TYR4	60.24
ACE2:GLU398 - VAL3 [hb]	99.40	ACE2:ARG518 - PHE10	99.90	ACE2:ALA348 - TRP6	95.10	ACE2:TYR510 - ARG8	53.45
ACE2:TYR515 - GLN8 [hb]	47.95	ACE2:ARG514 - TRP6	74.33	ACE2:ASN394 - LEU1	72.53	ACE2:ASP350 - ARG9	98.10
ACE2:PHE390 - TYR4 [sb]	63.14			ACE2:ARG518 - GLN8	84.12	ACE2:ASP382 - ARG9	48.85
ACE2:TYR510 - PRO5 [hb]	53.35			ACE2:TYR510 - PRO5	97.70	ACE2:GLU145 - TRP6	67.13
ACE2:ALA348 - THR7 [hb]	86.31			ACE2:HIS505 - THR7	56.54		
ACE2:ARG273 - ARG9 [hb]	66.33			ACE2:TYR515 - GLN8	96.10		
				ACE2:ASP509 - TYR4	89.81		
				ACE2:GLU375 - GLN8	100		

Table S5. The percentage of contact time during which intermolecular hydrophobic contacts were retained between ACE2 and the hemorphin peptides in the 100 ns simulation.

**Camel LVVHem6**		**LVVHem6**		**Camel LVVHem5**		**Camel LVVHem7**	
ACE2-LEU1		ACE2-LEU1		ACE2-LEU1		ACE2-LEU1	
TRP203	62.14	LEU391	100	ALA99	68.53	ALA153	99.10
TYR202	99.10	LEU73	97.70	TYR196	100	PHE274	60.94
ACE2-VAL2		PHE390	95.90	TYR202	83.52	ACE2-VAL2	
LEU391	60.64	TRP203	84.92	ACE2-PRO5		ALA153	82.42
PHE390	99.90	TRP69	92.81	LEU391	96.20	LEU144	99.50
PHE40	100	TYR202	99.40	PHE390	100	LEU148	88.01
TRP69	97.80	ACE2-VAL2		PHE40	100	MET152	100
ACE2-VAL3		LEU391	69.73	TYR385	75.72	MET270	100
PHE40	88.11	PHE390	96.90	ACE2-VAL2		TRP271	75.12
TYR202	76.52	PHE40	92.11	ALA99	100	ACE2-VAL3	
ACE2-TYR4		TRP203	53.15	LEU391	97.50	LEU144	95.40
TRP203	99.80	TRP69	99.30	LEU392	78.82	MET270	60.24
TYR510	100	TYR510	60.34	LEU95	91.71	PHE274	81.22
ACE2-PRO5		ACE2-VAL3		ACE2-TYR4		PRO346	97.10
ALA348	100	MET190	56.14	ALA99	93.11	TRP271	100
TRP349	100	TRP203	95.20	LEU100	91.01	ACE2-TYR4	
TYR510	100	TRP69	99.30	LEU391	100	CYS344	99.20
ACE2-TRP6		TYR199	99.60	LEU73	99.80	LEU143	75.92
ALA348	100	TYR510	99.10	PHE32	64.54	LEU144	100
ILE379	66.63	ACE2-TYR4		PHE390	100	PHE504	90.71
PRO346	98.70	LEU120	100	PHE40	98.90	PRO346	100
TRP349	99.80	PHE40	50.25	TRP69	96.40	TRP168	98.40
TYR385	93.61	TRP69	99.40	ACE2-TRP6		TRP271	100
TYR510	99.00	TYR510	100	ALA348	64.34	TYR127	96.90
		ACE2-PRO5		PHE40	59.64	VAL343	52.95
		PHE40	100	TRP349	89.61	ACE2-PRO5	

		TRP349	99.30	TYR510	70.93	LEU503	96.90
		TRP69	99.30			PHE274	65.93
		TYR510	70.03			PHE504	99.80
		ACE2-TRP6				PHE512	100
		ALA348	100			PRO346	99.30
		PHE40	59.14			TRP271	99.40
		PHE504	86.71			TYR515	100
		TRP349	100			ACE2-TRP6	
		TYR510	100			LEU370	100
						PHE274	94.51
						PRO346	100
						TYR515	100
						ACE2-PHE10	
						PHE40	68.63
						TRP349	70.53
						TYR510	91.91
**VVHem6**		**LVVHem7**		**LVVHem5**		**Camel Hem7**	
ACE2-VAL2		ACE2-LEU1		ACE2-LEU1		ACE2-TYR4	
TRP203	99.60	LEU120	97.90	ALA99	99.70	ALA153	95.10
TYR202	99.80	MET62	100.00	LEU391	100.00	LEU370	49.75
ACE2-VAL3		PHE504	99.80	LEU392	70.53	MET152	75.82
TRP203	87.41	TRP349	90.21	PHE390	100.00	MET270	76.62
TYR510	100.00	TYR50	78.52	PHE40	99.10	PHE274	100.00
ACE2-TYR4		ACE2-VAL2		TRP69	61.64	PRO346	54.45
LEU391	96.10	LEU120	50.05	ACE2-VAL2		TRP271	51.75
LEU392	78.62	TRP349	67.13	LEU73	93.51	ACE2-PRO5	
PHE390	98.90	TYR510	93.21	PHE390	69.83	LEU370	93.51
PHE40	99.70	ACE2-VAL3		PHE40	94.31	PHE274	99.30
TYR510	56.74	LEU120	85.01	TRP69	90.61	PRO346	68.53
ACE2-PRO5		PHE40	53.85	ACE2-VAL3		ACE2-TRP6	

ALA348	80.42	TRP69	100.00	PHE40	99.40	LEU144	100.00
PHE40	90.00	TYR510	90.51	ACE2-TYR4		LEU503	80.02
TRP349	98.80	ACE2-TYR4		LEU120	95.70	PHE274	53.85
TYR510	100.00	ALA99	51.65	TYR199	76.52	PHE504	81.52
ACE2-TRP6		LEU391	98.70	TYR510	100.00	PRO346	94.51
ALA348	100.00	LEU73	59.04	ACE2-PRO5		TRP271	64.04
PHE504	71.93	PHE390	100.00	ALA348	98.20	TYR127	72.23
PRO346	50.85	PHE40	100.00	PHE504	70.13	TYR515	64.74
TRP349	100.00	TRP69	100.00	TRP349	100.00	ACE2-PHE10	
TYR510	100.00	TYR510	96.50	TYR510	100.00	ALA348	100.00
		ACE2-PRO5		ACE2-TRP6		LEU351	95.00
		PHE40	94.21	ALA348	100.00	PHE40	91.01
		TYR510	98.60	ILE379	51.75	TRP349	100.00
		ACE2-TRP6		PRO346	99.40	TYR510	84.12
		ALA348	62.54	TRP349	100.00		
		PHE40	100.00	TYR510	97.90		
		PHE504	91.41				
		TRP349	91.41				
		TYR510	99.90				
		ACE2-PHE10					
		LEU370	99.20				
		PHE274	100.00				
		PRO346	99.50				
		TYR515	99.50				

Appendix

IC_{50} values of initial single-run peptide screening.

All the purchased peptides were screened in an initial run at 0, 10, 50, 100, 200, and 500 µM. Of the eighteen peptides purchased, Hem5, LVVHem4, and camel Hem6 did not dissolve during sample preparation.

S.No	IC_{50}	Hemorphin	Peptide sequence
1	14.916	LVVHem6	LVVYPWTQR
2	21.197	Camel LVVHem5	LVVYPWTR
3	25.806	Camel LVVHem7	LVVYPWTRRF
4	25.922	Camel LVVHem6	LVVYPWTRR
5	29.264	LVVHem7	LVVYPWTQRF
6	30.333	VVHem6	VVYPWTQR
7	36.736	LVVHem5	LVVYPWTQ
8	44.074	Camel Hem7	YPWTRRF
9	44.135	Camel VVHem6	VVYPWTRR
10	51.371	Camel VVHem7	VVYPWTRRF
11	63.105	VVHem7	VVYPWTQRF
12	78.751	Camel Hem5	YPWTR
13	80.196	Hem6	YPWTQR
14	95.129	Hem7	YPWTQRF
15	No IC50	Hem4	YPWT
16	—	Hem5	YPWTQ
17	—	LVVHem4	LVVYPWT
18	—	Camel Hem6	YPWTRR
